# An Energy-Independent Pro-longevity Function of Triacylglycerol in Yeast

**DOI:** 10.1371/journal.pgen.1005878

**Published:** 2016-02-23

**Authors:** Witawas Handee, Xiaobo Li, Kevin W. Hall, Xiexiong Deng, Pan Li, Christoph Benning, Barry L. Williams, Min-Hao Kuo

**Affiliations:** 1 Department of Cell and Molecular Biology, Michigan State University. East Lansing, Michigan, United States of America; 2 DOE-Plant Research Laboratory, Michigan State University. East Lansing, Michigan, United States of America; 3 Department of Plant Biology, Michigan State University. East Lansing, Michigan, United States of America; 4 Department of Integrative Biology, Michigan State University. East Lansing, Michigan, United States of America; 5 Department of Biochemistry and Molecular Biology, Michigan State University. East Lansing, Michigan, United States of America; University of Southern California, UNITED STATES

## Abstract

Intracellular triacylglycerol (TAG) is a ubiquitous energy storage lipid also involved in lipid homeostasis and signaling. Comparatively, little is known about TAG’s role in other cellular functions. Here we show a pro-longevity function of TAG in the budding yeast *Saccharomyces cerevisiae*. In yeast strains derived from natural and laboratory environments a correlation between high levels of TAG and longer chronological lifespan was observed. Increased TAG abundance through the deletion of TAG lipases prolonged chronological lifespan of laboratory strains, while diminishing TAG biosynthesis shortened lifespan without apparently affecting vegetative growth. TAG-mediated lifespan extension was independent of several other known stress response factors involved in chronological aging. Because both lifespan regulation and TAG metabolism are conserved, this cellular pro-longevity function of TAG may extend to other organisms.

## Author Summary

Triacylglycerol (TAG) is a ubiquitous lipid species well-known for its roles in storing surplus energy, providing insulation, and maintaining cellular lipid homeostasis. Here we present evidence for a novel pro-longevity function of TAG in the budding yeast, a model organism for aging research. Yeast cells that are genetically engineered to store more TAG live significantly longer without suffering obvious growth defects, whereas those lean cells that are depleted of TAG die early. Yeast strains isolated from the wild in general contain more fat and also display longer lifespan. One of the approaches taken here to force the increase of intracellular TAG is to delete lipases responsible for lipid hydrolysis. Energy extraction from TAG thus is unlikely an underlying cause of the observed lifespan extension. Our results are reminiscent of certain animal studies linking higher body fat to longer lifespan. Potential mechanisms for the connection of TAG and yeast lifespan regulation are discussed.

## Introduction

Lipid is essential for all life forms on earth. Polar lipids, most notably phospholipids, are the primary components of biological membranes, whereas neutral lipids such as triacylglycerols (TAG; or triglycerides, TG), are long believed to store excessive energy and to provide thermal and physical insulation for animals. By esterifying three molecules of fatty acids to the glycerol backbone, TAG packs the highest density of chemical energy among major biomolecules, consistent with a role in storing surplus energy. In addition, TAG metabolism is linked to the overall lipid homeostasis in cells and the organism [1]. This is commonly achieved via diacylglycerol, DAG, which is a shared precursor for TAG and phospholipids biosynthesis. Interestingly, in many organisms, TAG accumulation is not a response of surplus nutrients, but of different stresses. For example, starvation for nitrogen, phosphorus, or sulfur causes a model photosynthetic microalga *Chlamydomonas reinhardtii* to accumulate TAG [2, 3]. Even the oleaginous marine alga *Nannochloropsis* species increase the TAG content by 50% when starved of nitrogen, and to a lesser extent when stressed by high light and salinity [4]. In animals, mild (5%) calorie restriction administered to laboratory mice shifted the relative abundance of fat and muscle by increasing the fat mass by 68%, and reducing the lean mass by 12%, with total bodyweight remained unchanged [5]. In budding yeast *Saccharomyces cerevisiae*, a minimal amount of TAG is synthesized by vegetatively growing cells. When glucose becomes limited and that cells enter the stationary phase, TAG synthesis rises sharply [6]. One apparent reason for these organisms to maintain a larger storage of TAG under stress is to cope with the uncertainties of the environments. By storing chemical energy and materials for membrane lipid biosynthesis, TAG helps the underlying cells quickly resume robust metabolism and growth when conditions improve. On the other hand, whether the existence of TAG in cells affords other benefits for survival through the environmental stress remains an unaddressed question.

TAG is composed of a glycerol backbone esterified to three fatty acids by acyl coenzyme A:diacylglycerol acyltransferases, DGATs, and phospholipid:diacylglycerol acyltransferases, PDATs. In budding yeast, Dga1p and Lro1p are the major DGAT and PDAT, respectively [7]. Of these two, Lro1p appears to be responsible for TAG synthesis in vegetatively growing cells, whereas Dga1p contributes more significantly to the post-diauxic shift accumulation of TAG [8–10]. In addition to TAG, fatty acids can be esterified to sterols to form steryl esters (SE), another class of storage neutral lipids [10]. The two major enzymes responsible for SE biosynthesis are Are1p and Are2p [10]. In contrast to the stark differentiation of TAG abundance in log and stationary phase cells, SEs are maintained at a constant level at different phases of the growth curve [11], suggesting a function unique to TAG in the stationary phase. Intriguingly, deleting the four major neutral lipid biosynthetic genes (*DGA1*, *LRO1*, *ARE1*, *ARE2*), while causing yeast cells to lose practically all storage neutral lipids, does not result in significant deleterious effects in vegetatively growing cells [10]. However, these lean cells are hypersensitive to exogenous fatty acids and die with a phenotype of membrane over-proliferation [12], indicating that maintaining the capacity of incorporating excessive free fatty acids in the form of TAG or SE affords an important means to prevent lipotoxicity of free fatty acids. Accumulation of TAG and SE in the ER membrane causes expansion of the membrane, which eventually buds out to form lipid droplets (LD), a dynamic phospholipid monolayered organelle that has gained increasing research interests [13, 14]. A variety of proteins have been found associated with LD, including multiple TAG and SE hydrolases and signaling proteins that together play important roles in lipid homeostasis [15]. The yeast *TGL3* and *TGL4* genes encode the two major TAG lipases in yeast; additional lipases Tgl5p, Ayr1p and Lpx1p appear to be less robust enzymatically [16]. Tgl4p, which is thought to be the functional orthologue of the mammalian ATGL [17, 18], has been shown to be regulated by Cdk1-mediated phosphorylation in G1-to-S transition of dividing cells [19]. Blocking this phosphorylation event delays bud emergence. Deleting either or both major TAG lipases causes accumulation of TAG in stationary phase cells without a clear growth defect [18]. Similar to TAG, SE can be broken down by functionally redundant lipases Tgl1p, Yeh1p, and Yeh2p [20, 21]. SE lipase triple knockout cells, aside from possessing a significantly larger pool of SE, are phenotypically indistinguishable from the wildtype counterpart [20]. Together, these studies demonstrated clearly that yeast cells have the capacity of metabolizing neutral storage lipids. However, these lipids are not essential for cellular viability.

Budding yeast has been a model for two modes of aging [22, 23], chronological lifespan (CLS) and replicative lifespan (RLS). CLS refers to the overall viability of stationary-phase cells over time. RLS examines the number of daughters that each mother cell can produce before ceasing division. These two modes simulate, respectively, the senescence of post-mitotic (e.g., muscles and neurons) and stem cells in metazoans. Chronological aging in yeast has been linked closely to the nutrient status. When glucose is depleted, yeast cells exit from the log phase to enter diauxic shift, then into the stationary phase, a mitotically inactive yet metabolically active state [24]. The population viability is maintained in cells that enter the quiescent state [25, 26]. However, over time, the number of viable quiescent cells diminishes as well, resulting in a progressive increase of population mortality, a condition similar to metazoans including humans [22]. While there are yeast-specific chronological senescence and longevity factors (e.g., acetic acid and glycerol, respectively) [27, 28], a number of pathways are conserved [22, 23]. Some of the most notable pro-aging pathways include the Target Of Rapamycin (TOR)/S6 kinase (Sch9p in yeast) [29] and the Ras/adenylate cyclase/PKA pathways [30]. Intriguingly, these pathways control both CLS and RLS [23]. These two pathways are activated in response to the intake of selective nutrients, and are suppressed by caloric restriction, consistent with many reports that different organisms extend their lifespan when subjected to calorie restriction [31, 32]. Other pro-aging factors include oxidative stresses [33], mitochondrial dysfunction [34, 35], defective autophagy [36], DNA damages and replication stresses [37], and metabolic alterations [38]. Yeast genome-wide studies have identified a variety of gene mutations that extend chronological lifespan [39–42]. Many of these genes are involved in the metabolism of amino acids, nucleotides, or alternative carbon source, further underscoring the important roles played by metabolites in the control of lifespan. However, despite these relatively unbiased screens, very little is known regarding the role of lipids in the control of CLS. Here we present evidence for a novel energy usage-independent, anti-senescence function of TAG in yeast.

## Results

### TAG extends chronological lifespan

**Fig 1:**
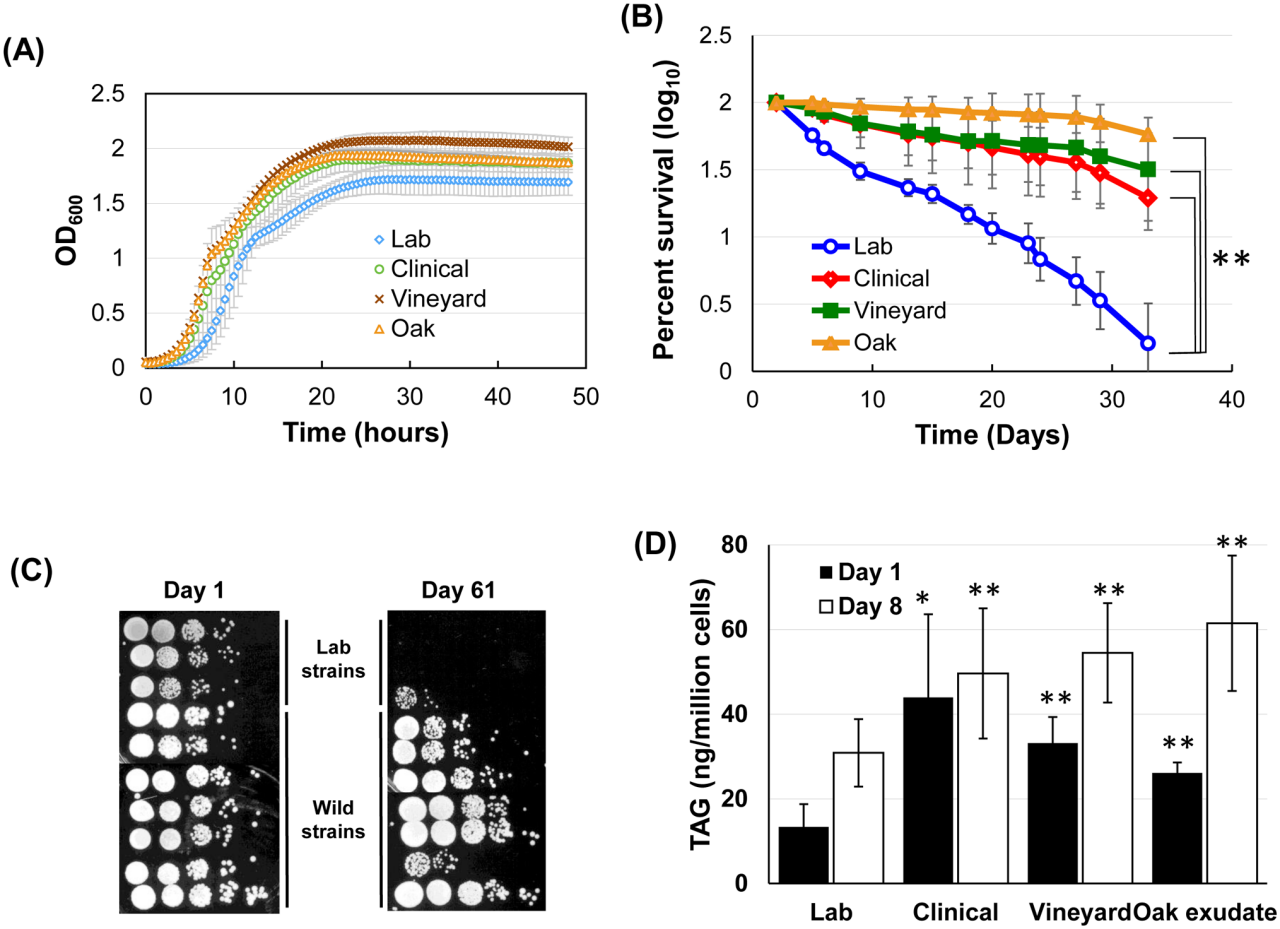
Wild yeast strains from different origins accumulate higher levels of triacylglycerol (TAG) and exhibit longer chronological lifespan. (A) Growth curves of wild strains isolated from oak exudates, vineyards, and clinical samples vs. three laboratory strains. Cells were grown in YPD at 30° in a 96-well plate. Each curve represents the average of growth of 2 to 4 strains in the category. Shown are representative results from two biological replicates. (B) Chronological lifespan in SC medium was measured by the “outgrowth” approach. (C) Chronological lifespan examined by spot assays. Viability of cultures was assayed every 3 to 5 days for two months. Only day 1 (all strains showed close to 100% viability) and day 61 images are shown. Cultures were 10-fold serially diluted in water before inoculation. (D) Quantification of triacylglycerol of cells harvested from day 1 and day 8 post-saturation YPD cultures.

Budding yeast is an excellent model for elucidating gene functions, biochemical pathways, stress responses, and aging. Natural variations among yeast strains, including traits altered during laboratory domestication may help uncover the relationships between genotype and phenotype [43, 44]. We speculated that phenotypic differences between laboratory and wild isolates could reveal important biological information, including the relationship between lipid and aging. To this end, we first examined the growth curves, under standard laboratory growth conditions, of eight wild strains isolated from diverse natural environments and three laboratory strains. These wild strains were *ho*^-^ (i.e., unable to switch mating type) haploid segregants of isolates from vineyards, oak exudates, and clinical samples. The three laboratory strains were yMK839, a derivative of EG123 [45], W303, and BY4742. Fig 1A shows that, in general, wild strains had a shorter lag phase and faster growth rate in log phase than laboratory strains (doubling time in YPD: lab, 94 ± 2 min; wild, 80 ± 3 min). In addition, the cell density at saturation, i.e., stationary phase, was also higher for the wild strains. The ability to accumulate higher cell density in spent medium indicated that these wild strains may have adapted more effectively to nutrient limitations in a harsh environment, and, if true, further suggested that such cells might survive better through stationary phase than their domesticated counterparts. To test this hypothesis, we measured the percent of cells able to re-enter vegetative growth from revival of the saturated cultures over a period of one month, using the quantitative “outgrowth” approach developed by Kaeberlein and colleagues [46]. The survival plot in Fig 1B demonstrates higher viability of wild strains. In a separate “spot assay”, cells cultivated for 61 days were serially diluted and spotted to a fresh solid medium. Two of the three laboratory strains fell below the detection limit of this assay, whereas the majority of the wild strains were capable of forming new colonies, indicating higher survival rates (Fig 1C).

Fig 1A–1C suggest that the laboratory domestication of *S*. *cerevisiae* might have artificially selected for certain physiology features leading to distinct phenotypes in the stationary phase [47]. Microscopic inspection of 5-day old stationary phase cells revealed more abundant cytoplasmic granules stainable by the neutral lipid dye Nile red in wild strains (S1 Fig). These Nile red stained lipid droplets are organelles that store neutral lipids, i.e., triacylglycerol and steryl esters [14]. Whereas the SE level stays constant through the growth curve [11], TAG abundance rises sharply when cells enter the stationary phase [6]. TAG thus seemed to be a plausible causal link to the observed difference in survival following saturation. To test this hypothesis, we first quantified cellular TAG abundance of day-1 and day-8 post-saturation cultures. Consistent with the microscopic observations, the wild strains contained higher levels of TAG at both time points (Fig 1D), providing a positive correlation between TAG abundance and survival.

The differential age-dependent survival shown above is equivalent to yeast chronological lifespan [48, 49], suggesting that TAG may have a role in maintaining or even extending chronological lifespan. To understand the causal relationship between TAG metabolism and chronological lifespan, we turned to a laboratory strain (yMK839) for genetic manipulation and phenotypic assessment.

**Fig 2:**
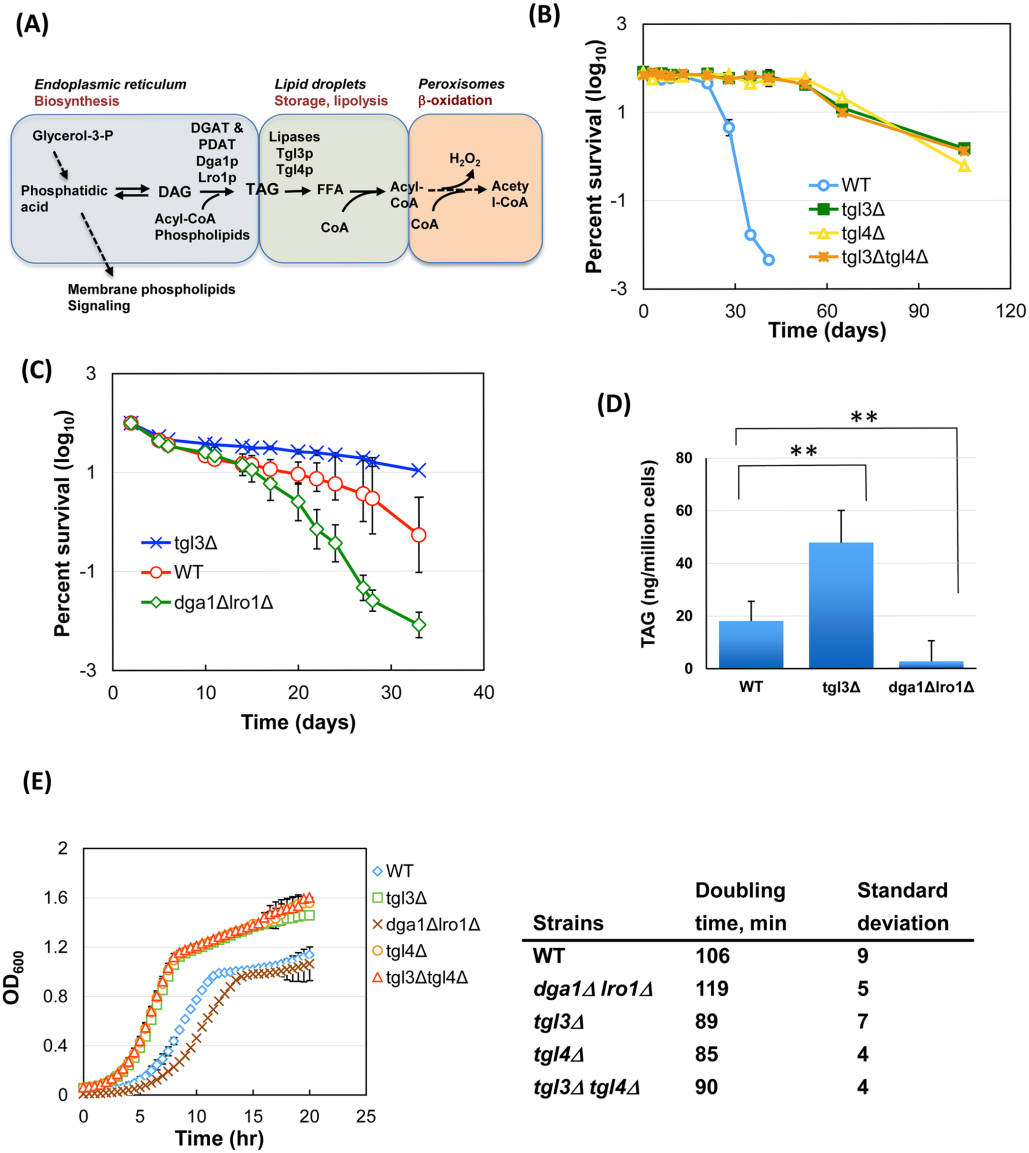
Elevated intracellular TAG level promotes longevity during chronological aging, whereas TAG depletion shortens lifespan. (A) Abbreviated view of TAG metabolism in yeast. (B) A laboratory strain yMK839 and its single and double lipase knockout derivatives were grown deeply into stationary phase in SC medium. At different time points, cells were spread to fresh YPD plates to quantify the colony forming units, expressed as percent survival. The plot was from two biological replicates of each strain. (C) Deleting *DGA1* and *LRO1* causes early death in stationary phase. Shown are averages of five biological repeats by the outgrowth approach. ** p < 0.01. Note the difference in time scale between panel B and panel C. Also, the two different assays for chronological lifespan quantification, i.e., colony forming units and outgrowth method, might give rise to differences in the absolute numbers of percent survival. The former did not differentiate colony size variations, whereas the latter outgrowth assay, which relied on the population growth rate, would be impacted by differences in doubling time and in the time when a cell exited the lag phase. (D) Quantification of intracellular TAG from day-8 post-saturation cultures in SC medium. (E) Growth comparison of yMK839 and its TAG-rich and -depleted derivatives. Growth curves were obtained as in Fig 1A.

In yeast, Dga1p and Lro1p are the major TAG biosynthetic enzymes (Fig 2A). The aliphatic chain of each constituent fatty acid contains chemical energy that can be converted to metabolically useful energy by a series of -oxidation reactions taking place in peroxisomes, following lipolysis by lipases Tgl3p and Tgl4p [18, 50, 51]. To test whether the increased TAG storage prolongs chronological lifespan, we deleted *TGL3* and *TGL4*, which caused TAG accumulation while blocking energy extraction from this lipid species. Consistent with published results [18, 51], the TAG level was elevated in *tgl3*Δ, *tgl4*Δ, and *tgl3*Δ *tgl4*Δ strains at stationary phase (S2 Fig). Importantly, all three TAG-rich strains showed extension of chronological lifespan (Fig 2B, and S5 Fig rows 2 to 4). These results indicate that the storage of TAG, but not its hydrolysis as a prerequisite for energy conversion or other cellular uses, is associated with improved viability in stationary phase. Intriguingly, deleting the TAG lipase did not cause discernible defects in doubling time (Fig 2E), mating efficiency of haploid cells, or sporulation of homozygous *tgl3*Δ*/tgl3*Δ cells (S3 Fig). Because deleting either or both TAG lipase genes resulted in similar phenotypes with respect to TAG accumulation and lifespan extension (Fig 2B), the *tgl3*Δ strain is representatively shown in further experiments below.

Blocking TAG hydrolysis also diminishes downstream reactions, including peroxisomal -oxidation that produces H_2_O_2_ that may cause oxidative stresses and cell death [52]. If a reduction of lipolysis-associated H_2_O_2_ production was solely responsible for the observed viability retention, deleting the two major TAG biosynthetic acyltransferases, Dga1p and Lro1p, would prevent TAG synthesis and the subsequent -oxidation (Fig 2A), and a similar beneficial effect on longevity would result as well. However, phenotypic analysis of the *dga1*Δ *lro1*Δ strain revealed the opposite. Deleting the two TAG biosynthetic acyltransferases caused a reduction of chronological lifespan (Fig 2C, and S5 Fig, row 6) and nearly eliminated cellular TAG (Fig 2D). The log phase growth rate also was reduced by the double deletion by approximately 12% (Fig 2E), which likely resulted from a defect in maintaining the cell’s replicative potential (see Fig 6 and below). This shortened chronological lifespan could not be rescued by deleting *TGL3* (S5 Fig, row 5), underscoring the necessity of keeping the physical presence of TAG to maintain cellular viability during stationary phase.

**Fig 3:**
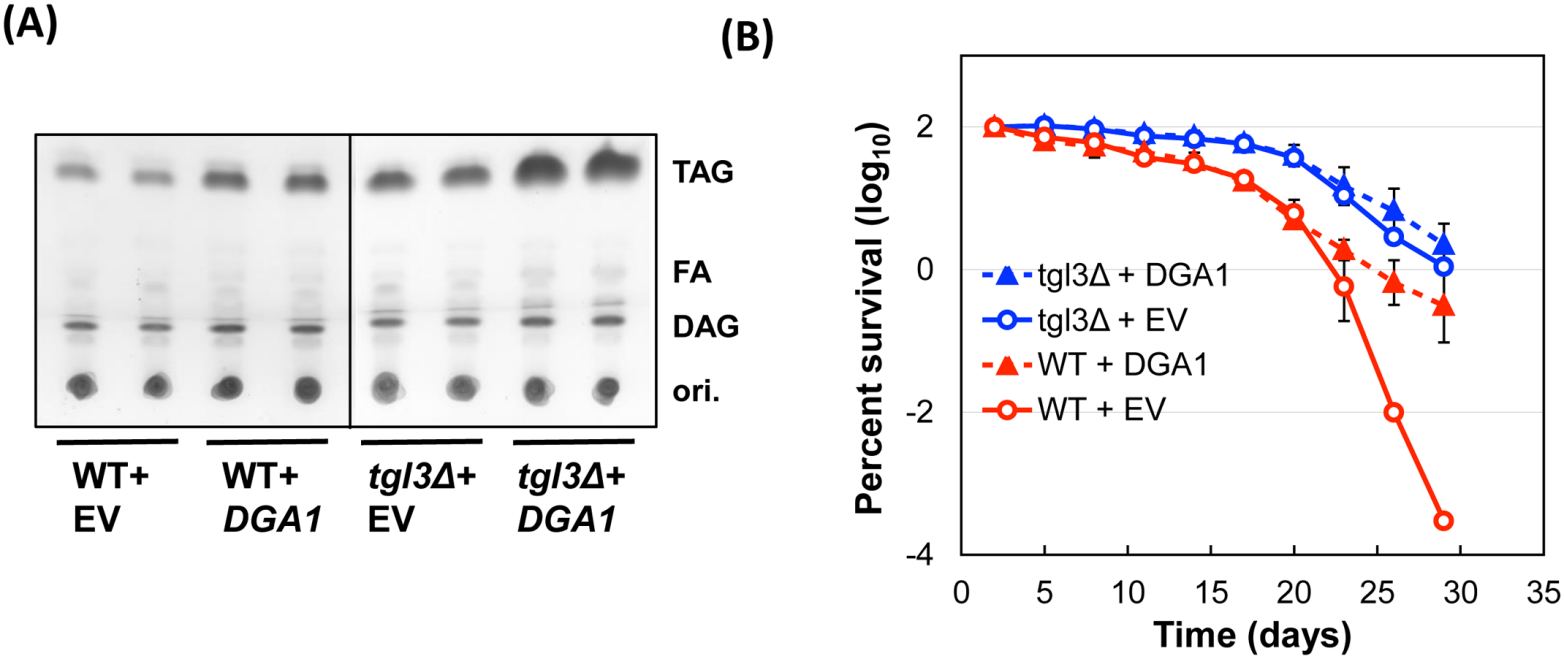
Overproducing Dga1p increases TAG abundance and extends chronological lifespan. (A) Thin-layer chromatography of neutral lipids from eight-day old stationary phase cultures bearing either the empty vector (EV) or one that expresses Dga1p. DAG, diacylglycerol; FA, free fatty acids; ori., origins for chromatography. The two EV lanes in panel A are biological duplicates; each of the two *DGA1* lanes represents the two different constructs with *DGA1* or *ADH1* promoter driving the recombinant gene expression. (B) Survival curves. These are averages of four *DGA1* overexpression isolates (two of each plasmid transformants), and two vector control duplicates. To accommodate the use of episomal plamids, yeast cells were grown in SC-uracil medium, which, compared with the use of synthetic complete medium seen in Figs 1 and 2, likely caused slightly faster viability loss of all strains analyzed.

To further confirm the pro-longevity role of TAG, we overexpressed a TAG biosynthetic enzyme Dga1p [9] by introducing a multi-copy plasmid bearing *DGA1* under the control of the native *DGA1* promoter or a constitutive *ADH1* promoter to *TGL3*^+^ and *tgl3*Δ strains. Cells from day-8 post-saturation cultures were processed for lipid extraction and TAG quantification. Data in Fig 3A confirmed the increased TAG content by Dga1p overproduction. The wildtype cells with a higher level of TAG exhibited longer lifespan (Fig 3B), strongly suggesting that TAG plays a causal role in preserving cellular viability during chronological senescence. Intriguingly, while Dga1p overexpression also raised the TAG content in *tgl3*Δ cells, the lifespan extension was relatively minor in this already long-living background. This observation indicates a limit of lifespan extension by TAG. Taking together the data in Figs 1 to 3, we conclude that intracellular triacylglycerol is essential for the maintenance of chronological lifespan, and that forcing the accumulation of TAG by either blocking its hydrolysis or increasing its biosynthesis, can extend lifespan.

### TAG promotes longevity independently of other lifespan control pathways

**Fig 4:**
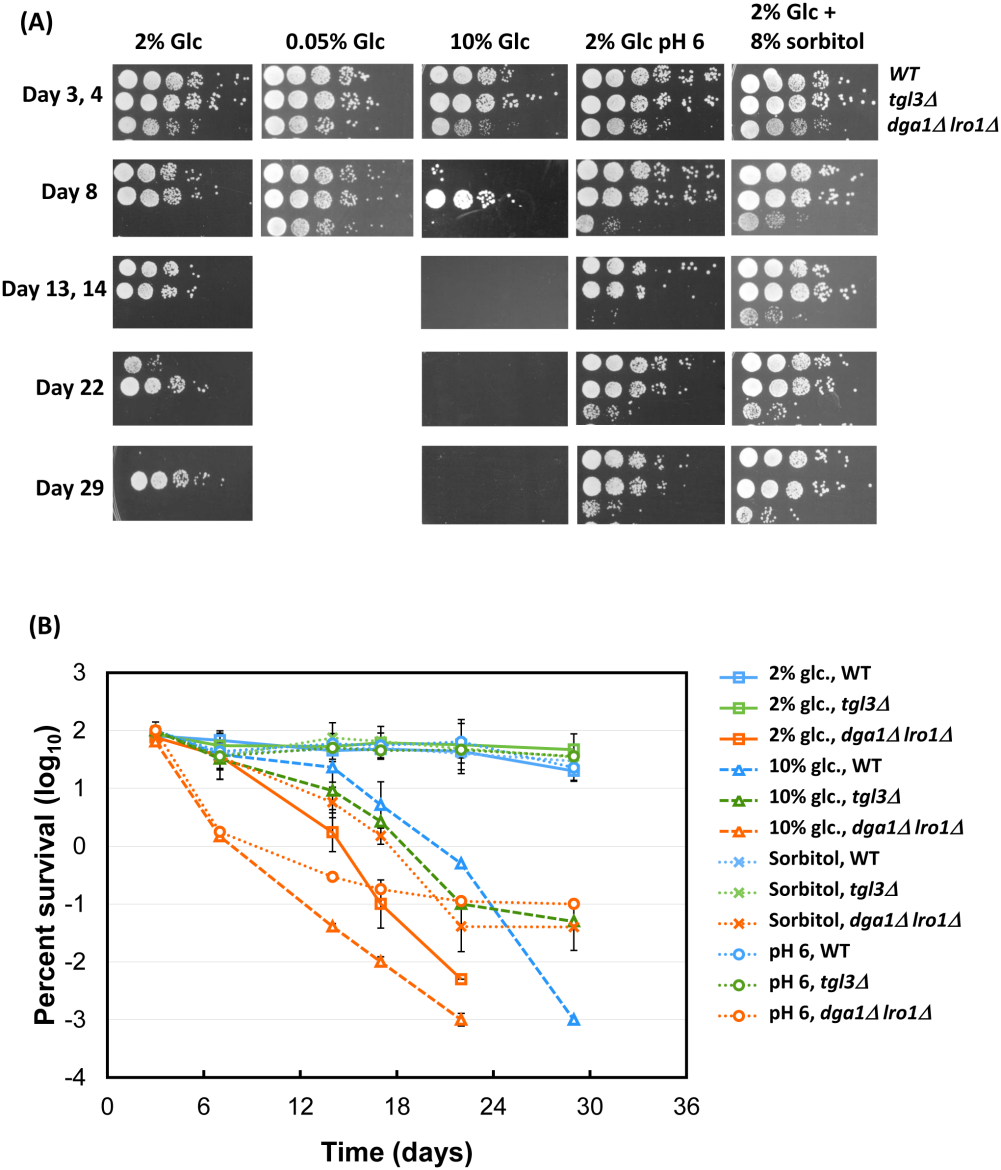
TAG-medicated lifespan control is independent of several yeast-specific and common lifespan regulatory regimes. (A) Semi-quantitative comparison of lifespan of the three “core” strains used in this study: wildtype (WT), TAG lipase knockout (*tgl3*Δ), and TAG synthesis deficient mutant (*dga1*Δ *lro1*Δ) under different growth conditions. Glc, glucose; the medium neutralization experiment (fourth column from left) was done with SC medium supplemented with 64.2 mM Na_2_HPO_4_ and citric acid to stabilize the pH at 6.0. Shown are representative results of 2 or 3 biological repeats. (B) Quantitation of the spot assay results. All data were from three biological duplicates.

**Fig 5:**
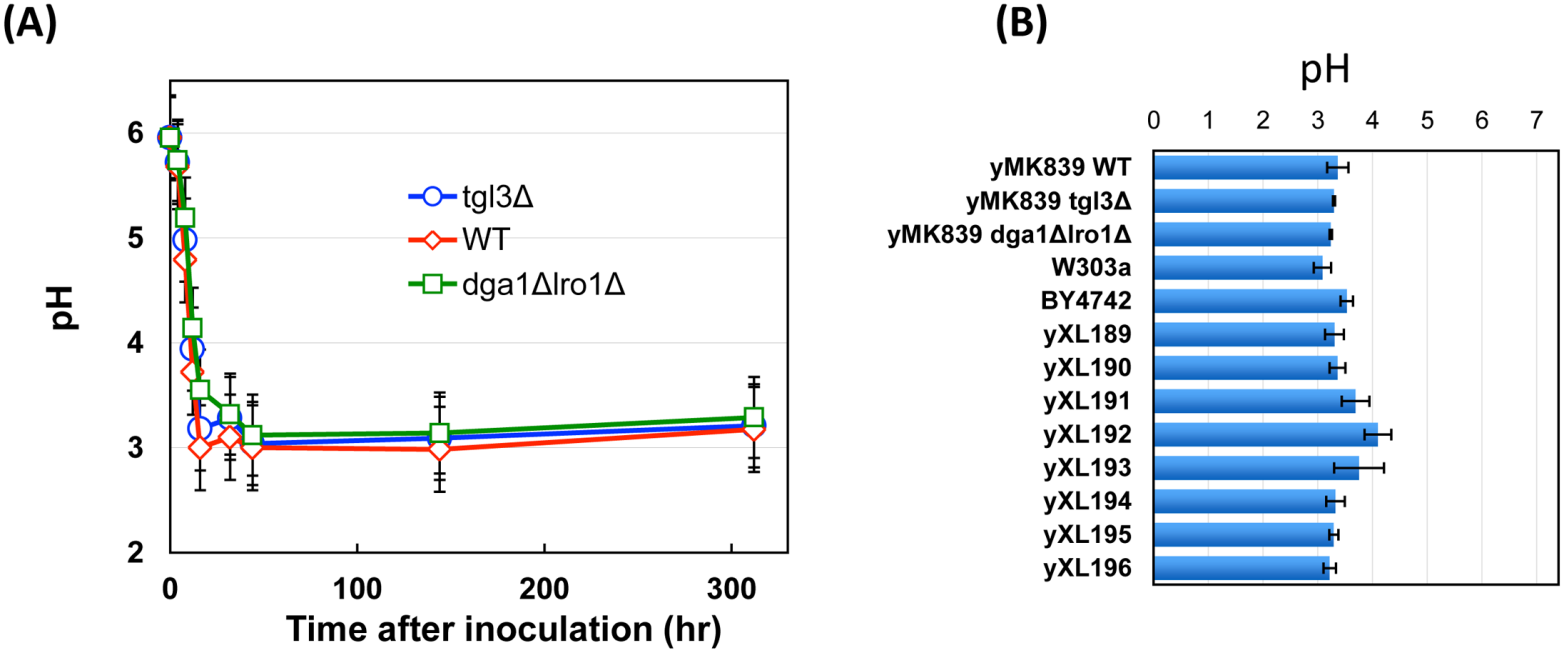
Culture medium pH changes are comparable among strains with different chronological lifespan. The pH changes of YPD cultures were monitored for 312 hours after inoculating overnight cultures to fresh medium (Panel A). In a second set of experiments (Panel B) the medium pH was measured from 4-day old YPD cultures of the indicated lab and wild strains. n ≥ 3.

The yeast chronological lifespan is regulated by common as well as yeast-specific factors. Rapamycin and paraquat extends and shortens lifespan, respectively [40, 53]. Caloric restriction, e.g., reducing the initial glucose concentration from 2% to 0.5% or lower in the medium, promotes longevity, whereas excessive glucose (e.g., 10%) shortens it. More specific to yeast is medium acidification from fermentation that causes senescence, an aging mechanism that can be antagonized by using a buffered, neutral pH medium [27, 54, 55]. We examined the relationship between TAG and these lifespan regulators, and found that the lifespan-extending regimes of caloric restriction (0.05% glucose), medium neutralization (pH6 with citrate phosphate), and high osmolarity (8% sorbitol) all delayed senescence for both the yMK839 wildtype and its TAG-depleted derivative (Fig 4A and 4B), with the latter still exhibiting shorter lifespan. The exceptionally long lifespan of *tgl3*Δ cells prohibited us from quantitatively assessing the effect of these lifespan extending treatments. 10% glucose caused all three strains to die early, yet the lipase-null cells remained to be the longest-living strain, suggesting strongly that CLS regulation by the abundance of TAG operates in a novel pathway. The observation that medium neutralization effectively extended the lifespan of *dga1*Δ *lro1*Δ and wildtype cells (Fig 4A fourth column from left) could be interpreted as that differences in medium acidification underlay the observed differential lifespan. However, direct measurement of the medium pH of the three normal, lean, and fat strains for more than 10 days (Fig 5A), or of 4-day old cultures of the three lab strains and 8 wild strains (Fig 5B) revealed statistically indistinguishable degrees of medium acidification. These data therefore ruled out that changes in the TAG level would alter the acidity of the medium and, consequently, the lifespan of cells. Together, Figs 4 and 5 demonstrate that the chronological lifespan can be controlled by the abundance of intracellular TAG in a mechanism that is independent of pathways involving glucose, medium pH and osmolarity.

**Fig 6:**
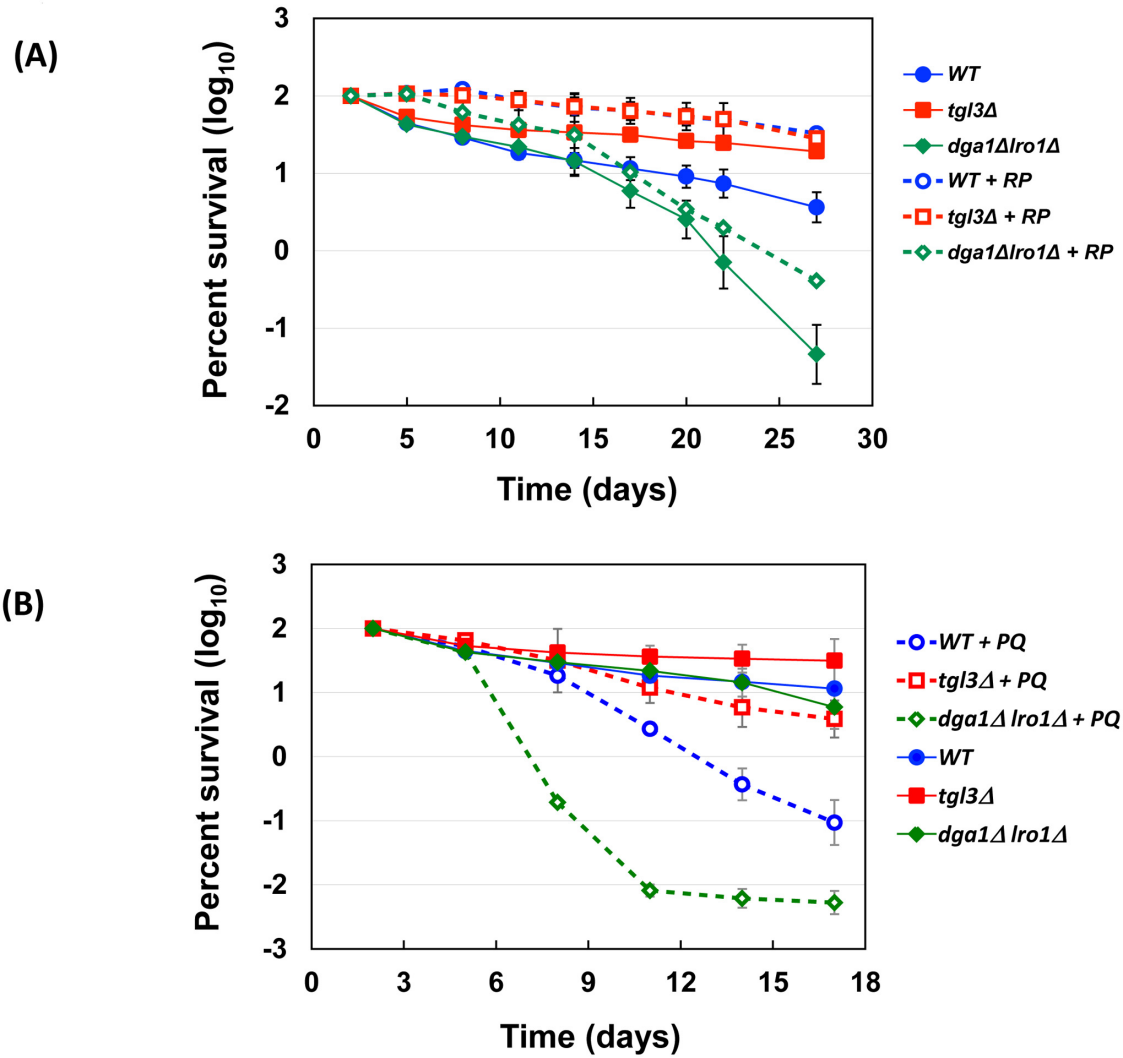
Rapamycin and paraquat respectively extends and shortens lifespan of the three core strains. 10 nM of Rapamycin (RP, panel A) and 10 μM of paraquat (PQ, panel B) were included in SC medium to assess the effects on the lifespan. Other parameters were identical to experiments shown in Fig 2C.

**Fig 7:**
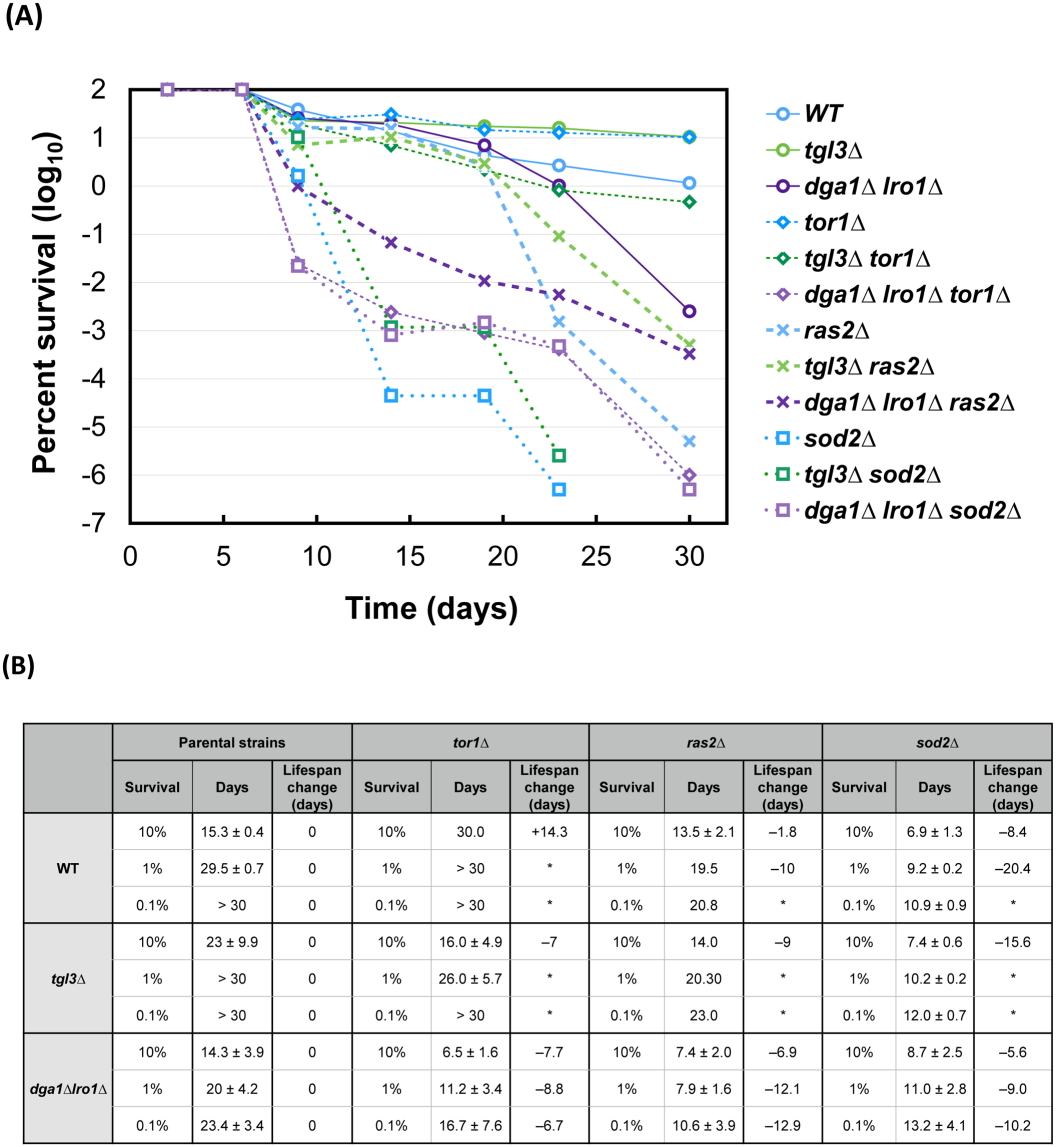
TAG controls chronological lifespan independently of conserved pathways. *TOR1*, *RAS2*, and *SOD2* were deleted from the three core strains with a normal, higher, and lower TAG content. The resultant strains were grown for outgrowth assays to compare their CLS with the corresponding parental strains. (A) Representative plot of one of three biological duplicate outgrowth experiments. (B) Quantitative analysis of lifespan from the outgrowth data seen in panel A. The time (in days) it took for each strain to drop to 10%, 1%, and 0.1% viability was calculated (see Materials and Methods) from two or three independent assays, whenever data available. Some thus did not yield a standard deviation. The lifespan changes of the *tor1*Δ, *ras2*Δ, and *sod2*Δ strains were differences (days to reach 10%, 1%, and 0.1% viability) from the corresponding parental strains. Asterisks (*) indicate that at least one of the two strains being compared maintained the viability higher than 1% or 0.1% throughout the duration of the experiments.

Rapamycin and paraquat are two potent extragenic lifespan modulators for many species [31, 40]. When treated with these two compounds, all three core strains responded similarly. That is, rapamycin extended, whereas paraquat shortened the lifespan of all three (Fig 6). When several highly conserved lifespan control genes *TOR1*, *RAS2*, and *SOD2* were deleted from the three core strains, we observed differential responses (Fig 7A). From the time for each strain to drop to 10%, 1%, and 0.1% viability (Fig 7B), it is clear that deleting *TOR1* made the wildtype strain live longer, in agreement with previous findings [28]. Intriguingly, despite that rapamycin (10 nM) treatment prolonged cellular survival (Fig 6A), deleting the entire Target of Rapamycin *TOR1* gene actually shortened the lifespan of both the fat, *tgl3*Δ cells and the lean, *dga1*Δ *lro1*Δ cells (Fig 7). Because these lipase- and DGAT-deficient strains were unable to extract energy from TAG metabolism, we suspect that *tor1*Δ cells survived at least partly on the energy stored in TAG. Lacking either *TGL3* or *DGA1* and *LRO1* resulted in the loss of viability during chronological aging. In contrast to the differential effects *TOR1* deletion, knocking out *RAS2* or *SOD2* shortened lifespan of all three parental strains (crosses and open squares, Fig 7A). *RAS2* in the RAS/cAMP/PKA pathway is involved in stress response and lifespan control [56]. Deleting *RAS2* has been shown to preserve chronological lifespan [28]. However, a genome-wide screen showed reduced survival of chronologically aging *ras2*Δ cells [57]. In our hands, all *ras2*Δ strains died earlier than their corresponding parental strains regardless of the TAG content (Fig 7A, cross markers, and Fig 7B summary), suggesting that Ras2p controls the lifespan in a TAG-independent manner. Similarly, deleting *SOD2*, which encodes a mitochondrial manganese superoxide dismutase that is a key to the defense against reactive oxygen species originated from mitochondria, and to the preservation of full lifespan potential [58], significantly reduced the life expectancy of all three strains. These observations suggest that Sod2p remained to be a critical vitality enzyme in the long-living *tgl3*Δ cells, and that TAG likely functions independently of Sod2p to protect aged cells.

### TAG is required for achieving full replicative potential

**Fig 8:**
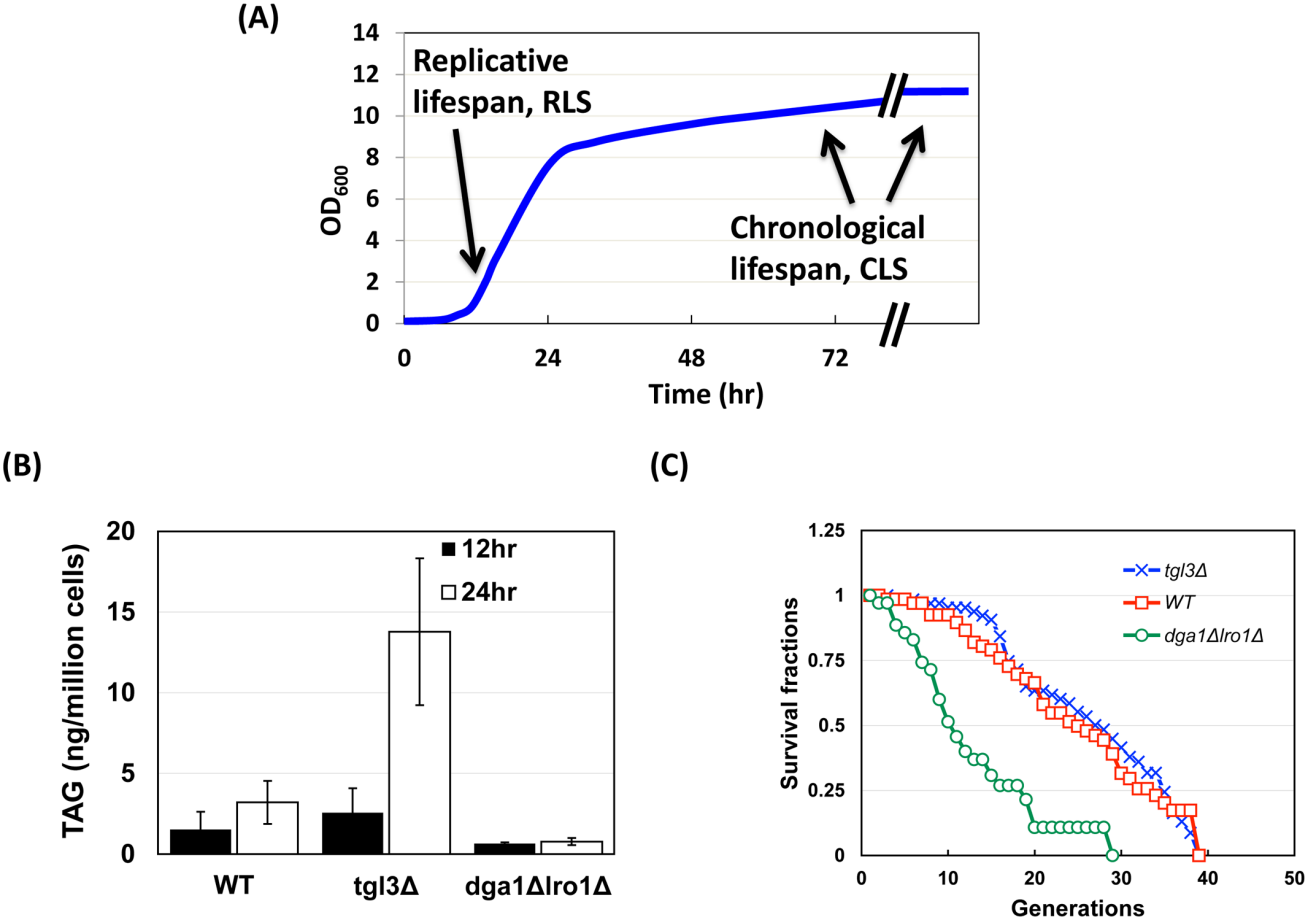
Full replicative lifespan requires TAG. (A) Replicative and chronological lifespan examine cells from log and stationary phase, respectively. (B) TAG quantification of strains harvested 12 and 24 hours after inoculation. These cells were at early and late log phase, respectively. The y axis was set at between 0 to 80 ng/million cells to be consistent with other similar Figs and to highlight the low abundance of TAG in log phase cells. (C) 60 to 90 newly divided daughter cells from the indicated strains were compared for their replicative lifespan. Shown are representative results from three biological duplicates. Breslow analysis showed that the RLS of the *dga1*Δ *lro1*Δ cells was significantly different from the other two strains (p < 0.0001).

Like CLS, replicative lifespan of budding yeast is another model for cellular senescence, which is determined as the number of daughters produced by a mother cell during its lifespan [48]. One fundamental difference between chronological and replicative lifespan is that the ability to proliferate is measured from cells sampled from saturation versus logarithmic growth, respectively (Fig 8A). Intriguingly, log phase yeast cells, which allocate most fatty acids to phospholipid synthesis to support cell growth and division, store very little TAG [6]. Although TAG is dispensable for cell survival [10] (Fig 2), the immediate precursor for TAG, diacylglycerol, also supplies building blocks for phospholipids [59]. It is possible that changes in the flux of the albeit small amount of TAG in dividing cells may still impact their replicative lifespan by, for example, influencing the metabolism of other lipids derived from DAG.

To assess the influence of TAG on replicative lifespan, yMK839 wildtype, *dga1*Δ *lro1*Δ, and *tgl3*Δ strains were subjected to replicative lifespan comparison by the traditional microscopy approach [23]. Deleting the TAG biosynthetic enzymes further diminished the small TAG pool in log phase cells (12-hour post-inoculation, black bars, Fig 8B). Importantly, both maximum and median replicative lifespan were decreased in cells depleted of TAG (Fig 8C). This shortened replicative lifespan likely accounted for the increased population doubling time of *dga1*Δ *lro1*Δ cells (Fig 2E). On the other hand, deleting *TGL3* had a minimal effect on the TAG level in early log phase cells (12-hour post-inoculation), and the lifespan of *tgl3*Δ cells also was unchanged (blue cross marker, Fig 8C). Together, these data demonstrate that maintaining a certain amount of TAG, or the ability to synthesize TAG, is required to reach full replicative potential. TAG hydrolysis apparently is not essential for replicative lifespan maintenance.

## Discussion

Here we present evidence for a novel pro-longevity function of intracellular TAG in yeast. Deleting TAG lipases or overproducing a DGAT increased TAG accumulation and extended chronological lifespan. Deleting the two TAG biosynthetic enzymes practically eliminated TAG and significantly shortened the chronological lifespan, as well as the median and maximum replicative lifespan. The fact that chronological lifespan extension is seen in different lipase knockout (i.e., *tgl3*Δ, *tgl4*Δ, and *tgl3*Δ *tgl4*Δ) and in DGAT overexpression strains argues strongly that the accumulation of TAG was the contributing factor for lifespan extension. This conclusion is consistent with the observation that deleting *TGL3* cannot rescue the early death phenotype of *dga1*Δ *lro1*Δ lean cells (rows 5 and 6, S5 Fig). Unlike other lifespan extension regimes such as *SCH9* knockout and rapamycin treatment that also retard mitotic growth [60, 61], we have yet to detect obvious growth defects in the three lipase deletion strains. For example, aside from normal, or even faster growth rates (Fig 2E), mating and sporulation efficiency of these fat cells was essentially identical to their wildtype mother strain (S3A and S3B Fig). There were no significant differences in cellular sensitivity to heat (55°C), 260 nm UV, high concentrations of NaCl, or to H_2_O_2_ (S4 Fig). It is perceivable that a negative phenotype would be linked to TAG lipase null cells if they were grown without any fatty acid supplement, and with concomitant presence of a fatty acid synthase inhibitor such as cerulenin [62]. These cells would suffer from the lack of energy and fatty acid building blocks for growth and division. TAG hydrolysis is thus by and large dispensable as long as fatty acids are available from the environment or can be synthesized by de novo activities. It should be noted that Daum and colleagues reported that *tgl3*^-^ homozygous knockout cells were unable to form spores [18]. Possible causes for this discrepancy may include differences in the genetic background of the strains, and the protocols for sporulation.

Chronological lifespan of *S*. *cerevisiae* is regulated by both yeast-specific and conserved factors and drugs. Experimental results shown in Figs 4 to 7 strongly suggest that TAG preserves viability during chronological senescence in a manner that is independent of those factors tested herein, including caloric restriction, high osmolarity, medium pH, rapamycin and paraquat responses, and conserved pathways involving *TOR1*, *RAS2*, and *SOD2* genes. Results from genetic interaction tests also suggest a novel TAG pathway in CLS control. Both fat and lean strains responded similarly to the deletion of *TOR1*, *RAS2*, or *SOD2* (Fig 7), indicating that changes in TAG metabolism does not affect the function of these conserved CLS regulators. Intriguingly, deleting *TOR1* shortens the lifespan of both lipase-null and DGAT-deficient cells (Fig 7) but, as expected, protects those cells possessing the normal TAG metabolic capacity. We suggest that *tor1*Δ cells that survive on the caloric restriction pathway through chronological aging [23, 28] need to tap into the energy depot of TAG. Without TAG biosynthetic enzymes or TAG hydrolytic lipases perturbs this energy flux, thus resulting in early death of *tor1*Δ cells. In addition to *TOR1* and *RAS2*, we have also combined *tgl3*Δ and *sch9*Δ mutations. Deleting *SCH9*, the ribosome S6 kinase homologue, has been shown to activate the Rim15-Msn2/4 and superoxide dismutase (SOD) stress pathways and prolongs lifespan significantly [29, 63]. Our tests of the genetic interaction between *TGL3* and *SCH9* were inconclusive. Independent *tgl3*Δ *sch9*Δ isolates showed mixed results, ranging from longer CLS to synthetic sickness (S6 Fig). The reason for the stochastic phenotypes is unclear.

Taking into account the observations presented above as well as from previous reports, we hypothesize that TAG has a role in stress response that underlies the observed phenotypes in chronological aging. Firstly, TAG accumulates when yeast cells enter stationary phase in which nutrients are becoming progressively limited [6, 47]. Starvation and stress-induced TAG accumulation appears to be a widespread response in different organisms, including photosynthetic algae [2–4, 64] and animals as well [65–67]. Dietary restriction has been suggested to prolong lifespan by eliciting cellular stress response [31]. Intriguingly, laboratory mice [5] and developing *Caenorhabditis elegans* [68] have an increased body fat mass when subjected to dietary restriction. Secondly, wild yeast strains in general exhibit higher TAG content and longer chronological lifespan (Fig 1). Food shortage is a common environmental crisis in the wild, but rarely a relevant factor for lab strains. A systematic phenotypic and transcriptomic survey of wild and laboratory strains showed that the latter are less tolerant of many environmental stresses [44]. Certain traits, including stress responses and high levels of TAG, might have lost during the domestication of *S*. *cerevisiae* in laboratory environments, in which the selection pressure for long-living, stress-tolerant stationary phase cells is low. It seems plausible that besides preserving energy to cope with uncertainties in food supply, the increased fat content in stressed cells may confer an additional, energy-independent function that helps sustain longevity.

The presumptive stress antagonized by TAG in post-mitotic cells remains to be identified. One candidate is fatty acid-solicited lipotoxicity [69]. Sequestering fatty acids in the form of TAG may prevent lipotoxicity that erodes replicative potential and chronological viability. Disabling TAG biosynthesis results in surplus fatty acids, which may arise from de novo synthesis, uptake from environment, or from lipolysis, that may disrupt membrane lipid homeostasis [9]. Indeed, in an extreme situation where the ability to incorporate fatty acids to TAG and SE is altogether eliminated, yeast cells become hypersensitive to fatty acids and die with membrane hyper-proliferation [12]. Similarly, the fission yeast *Schizosaccharomyces pombe dga1*^+^ and *plh1*^+^ double knockout cells (equivalent to the *dga1*Δ *lro1*Δ strain of *S*. *cerevisiae*) also die upon entering stationary phase, and are hypersensitive to exogenous fatty acids during vegetative growth [70]. While this lipotoxicity model explains the early death phenotype of *dga1*Δ *lro1*Δ cells, total lipid analysis of our long-living fat cells failed to detect significant changes in free fatty acids or DAG (see, for example, Fig 3A). While we cannot rule out the possibility that a small but critical change in certain lipid species contributes more critically to lifespan extension, other hypotheses are worth considering.

One frequently cited cause of aging is mitochondrial dysfunction that also involves oxidative damages [71]. While subcellular compartmentalization confines TAG synthesis and storage to ER and lipid droplets, respectively, a number of reports have demonstrated physical association of mitochondria with ER and LD [72], lending support for functional crosstalk between neutral lipid metabolism and mitochondria biogenesis [73]. Moreover, mitochondria also possess a type II fatty acid synthesis pathway [74]. Deleting enzymes within this pathway causes mouse embryonic lethality and yeast respiratory defects [75, 76]. Fatty acids trafficking between mitochondria and LD may help achieve mitochondrial lipid homeostasis. Importantly, mitochondria are a major source for reactive oxygen species. Free radicals that would otherwise escape from mitochondria and cause pleiotropic cellular damages might enter LD and attack the fatty acyl chains of the storage TAG molecules [77]. It is possible that the high density of peroxidated fatty acids in LD facilitates crosslinking of neighboring radicalized molecules, hence terminating the vicious propagation of radicals. This “radicals sink” model appears to be consistent with the experimental findings presented above.

TAG metabolism and many aspects of cellular aging are conserved. It is thus possible that the cytoprotective role of TAG also exists in higher organisms. For example, Bailey *et al*. recently reported an anti-oxidant role of lipid droplets in the stem cell niche of *Drosophila* during neurodevelopment by limiting the levels of reactive oxygen species and inhibiting the oxidation of polyunsaturated fatty acids [78]. In transgenic mice, overexpression of DGAT1 in the skeletal muscle and heart increased intracellular TAG abundance as well as insulin sensitivity of the underlying animals [79, 80]. Similarly, deleting adipose triglyceride lipase (ATGL) protected animals from high-fat diet-induced insulin resistance [81]. However, excessive TAG in heart muscle resulting from ATGL knockout also was associated with cardiac dysfunction [73, 82]. These transgenic animal studies underscore the complexity of mammalian metabolism and the interdigitating relationships between triglycerides (dietary, circulating, and in different tissues) and other nutrients. While the positive influence of intracellular TAG on chronological lifespan in yeast is reminiscent of the so-called obesity paradox in humans, that is, the overweight population has the lowest mortality under a number of medical conditions [83, 84], we caution that the comparatively simple yeast may not be immediately applicable to the complex human system. An integrative strategy combining metabolomics, lipidomics, and transcriptomics of representative yeast strains will help elucidate the molecular basis of this novel function of TAG, which might provide a toolbox for a better understanding of the benefits of intracellular TAG in humans.

## Materials and Methods

### Strains and media

Yeast strains used in this study are shown in Table 1. YPD medium contained 2% glucose (Sigma-Aldrich) (unless otherwise stated in the text), 2% peptone (BD Difco), and 1% yeast extract (BD Difco). SC medium (synthetic complete) contained 2% glucose, 5 g/l ammonium sulfate (Sigma-Aldrich), 1.7 g/l yeast nitrogen base without amino acids or ammonium sulfate (BD Difco), and complete amino acids as described in [85]. Auxotrophic nutrients were supplied at four-fold excess as recommended [23]. Citrate phosphate buffering was done as described [27]. Rapamycin (Sigma-Aldrich) and paraquat dichloride (Fluka) were added from 10 μM and 250 mM stocks to SC medium to make final concentrations of 10 nM and 10 mM, respectively.

**Table 1:** Yeast strains used in this study.

Strains	Relevant genotypes	Sources or references
yMK839	*MATa leu2-3 trp1 ura3-52*	[45]
yXL004	From yMK839 *MATa leu2-3 trp1 ura3-52 tgl3*Δ:: *TRP1*	[2]
yXL001	From yMK839 *MATa leu2-3 trp1 ura3-52 tgl4*Δ:: *TRP1*	[2]
yXL005	From yMK839 *MATa leu2-3 trp1 ura3-52 tgl3*Δ:: *KanMX; tgl4*Δ:: *TRP1*	[2]
yXL172	From yMK839 *MATa leu2-3 ura3-52 dga1*Δ:: *KanMX; lro1*Δ:: *URA3*	This study
yWH001	From yMK839 *MATa leu2-3 trp1 ura3-52 tgl3*Δ:: *TRP1; dga1*Δ:: *KanMX; lro1*Δ:: *URA3*	This study
yXL023	From yMK839 *MATa leu2-3 trp1 ura3-52 pMK595 [2μ URA3]*	[2]
yXL041	From yMK839 *MATa leu2-3 trp1 ura3-52 tgl3*Δ:: *TRP1 pMK595 [2μ URA3]*	This study
yXL183	From yMK839 *MATa leu2-3 trp1 ura3-52 pMK595-DGA1 [2μ URA3]*	This study
yWH083	From yMK839 *MATa leu2-3 trp1 ura3-52 pMK595 P*_*DGA1*_*-DGA1 [2μ URA3]*	This study
yXL180	From yMK839 *MATa leu2-3 trp1 ura3-52 tgl3*Δ:: *TRP1 pMK595-DGA1 [2μ URA3]*	This study
yWH087	From yMK839 *MATa leu2-3 trp1 ura3-52 tgl3*Δ:: *TRP1 pMK595 P*_*DGA1*_*-DGA1 [2μ URA3]*	This study
W303a	*MAT*a *leu2-3*,*112 trp1-1 can1-100 ura3-1 ade2-1 his3-11*,*15*	
BY4742	*MAT*α *his3*Δ *1 leu2*Δ *0 lys2*Δ *0 ura3*Δ *0*	
yWH47	BY4742 with *tgl3*Δ:: *URA3*	This study
EJ72	*MATa/α gal*^-^ *trp1 leu2 ura3-52 his4*	[45]
yWH036	EJ72 with *tgl3Δ*:: *URA3/TGL3*	This study
yWH74	EJ72 with *tgl3Δ*:: *TRP1/tgl3Δ*:: *TRP1*	This study
70‹	*MAT‹ thr3 met*	[96]
227a	*MATa lys1*	[96]
yXL189 (B454‹; clinical sample)	*MAT‹ ho*::*Hygromycin*	This study. Haploid isoform of YJM454 from J. McCusker
yXL190 (B653‹; clinical sample)	*MAT‹ ho*::*hisG lys2 gal2*	This study. YJM128 [
yXL191 (B756‹; clinical sample)	*MAT‹ ho*::*KanMX6*	This study. Haploid isoform of YJM450 from J. McCusker
yXL192 (B779‹; clinical sample)	*MAT*‹ *ho*::*KanMX6*	This study. Haploid isoform of YJM326 from J. McCusker
yXL193 (B359‹; vineyard)	*MAT*‹ *ho*::*KanMX6*	This study. Haploid isoform of YCD51-4 from Burgundy region of France, 1948
yXL194 (B370‹; vineyard)	*MAT*‹ *ho*::*KanMX6*	This study. Haploid isoform of M5-2 from Italy in 1993 by R. Mortimer
yXL195 (B357‹; oak exudate)	*MAT*‹; *ho*::*KanMX6*	This study. Haploid isoform of YPS1009-2 from Mettler’s Woods, New Jersey in 2000 by P. Sniegowski
yXL196 (B390‹; oak exudate)	*MAT*‹ *ho*::*KanMX6*	This study. Haploid isoform of YPS1000-1 from Mettler’s Woods, New Jersey in 2000 by P. Sniegowski
yXD188	From yMK839 *MATa leu2-3 trp1 ura3-52 tor1*Δ:: *URA3*	This study
yXD189	From yMK839 *MATa leu2-3 trp1 ura3-52 ras2*Δ:: *URA3*	This study
yXD190	From yMK839 *MATa leu2-3 trp1 ura3-52 sod2*Δ:: *URA3*	This study
yXD191	From yMK839 *MATa leu2-3 trp1 ura3-52 tgl3*Δ:: *TRP1; tor1*Δ:: *URA3*	This study
yXD192	From yMK839 *MATa leu2-3 trp1 ura3-52 tgl3Δ*:: *TRP1; ras2Δ*:: *URA3*	This study
yXD193	From yMK839 *MATa leu2-3 trp1 ura3-52 tgl3Δ*:: *TRP1; sod2Δ*:: *URA3*	This study
yXD200	From yMK839 *MATa leu2-3 trp1 ura3-52 dga1Δ*:: *KanMX; lro1Δ*:: *TRP1*	This study
yXD201	From yMK839 *MATa leu2-3 trp1 ura3-52 dga1Δ*:: *KanMX; lro1Δ*:: *TRP1; tor1*Δ:: *URA3*	This study
yXD202	From yMK839 *MATa leu2-3 trp1 ura3-52 dga1Δ*:: *KanMX; lro1Δ*:: *TRP1; ras2*Δ:: *URA3*	This study
yXD203	From yMK839 *MATa leu2-3 trp1 ura3-52 dga1Δ*:: *KanMX; lro1Δ*:: *TRP1; sod2*Δ:: *URA3*	This study

### Yeast methods

Yeast transformation was performed using the lithium acetate method [86]. Deletion of *TGL3* and *TGL4* was described in [2]. To delete *DGA1*, PCR reactions using primers ATGTCAGGAACATTCAATGATATAAGAAGAAGGAAGAAGGAAGATCCCCGGGTTAATTAA and TTACCCAACTATCTTCAATTCTGCATCCGGTACCCCATATTTATTCGAGCTCGTTTAAAC and template pFA6a-KanMX6 [87] were conducted. To delete *LRO1*, primers TATCCATATGACGTTCCAGATTACGCTGCTCAGTGCG*GCCGC*ATGTCAGGAACATTCAAT and GAATTTCGACGGTATCGGGGGGATCCACTAGTTCTAGCTAGATTACCCAACTATCTTCAA were used with pBS1539 as the template to amplify *K*. *lactis URA3* gene as the selective marker [88]. To delete *TOR1*, primers GAACCGCATGAGGAGCAGATTTGGAAGAGTAAACTTTTGAAATGAAGCTTGATATCGAAT and CCAGAATGGGCACCATCCAATATAATGTTGACATAACCTTTCTACGACTCACTATAGGGC were used with pBS1539 to amplify *K*. *lactis URA3*. For *RAS2* deletion, primers CCTTTGAACAAGTCGAACATAAGAGAGTACAAGCTAGTCGTCTGAAGCTTGATATCGAAT and ACTTATAATACAACAGCCACCCGATCCGCTCTTGGAGGCTTCTACGACTCACTATAGGGC were used to amplify *K*. *lactis URA3* on pBS1539. For *SOD2* deletion, primers TTCGCGAAAACAGCAGCTGCTAATTTAACCAAGAAGGGTGGTTGAAGCTTGATATCGAAT and GATCTTGCCAGCATCGAATCTTCTGGATGCTTCTTTCCAGTTTACGACTCACTATAGGGC were used to amplify *K*. *lactis URA3* on pBS1539. The PCR products were gel-purified for yeast transformation. Genomic PCR was used to verify the correct insertion. To overproduce Dga1p, a yeast genomic PCR product was co-transformed with *Not* I-linearlized pMK595 [89] for *ADH1*-controlled expression (pMK595-*DGA1*). A second multicopy *DGA1* overexpression plasmid with *DGA1* under its own promoter control (pMK595 P_DGA1_-DGA1) was constructed similarly, except that the *DGA1* promoter (961 bp) was included in the transforming PCR DNA, using primers ATCCATATGACGTTCCAGATTACGCTGCTCAGTGCGGGTAAAGAATCTAAATCGAGCTAC and atcggggggatccactagttctagctagagcggccTAGATAGGTACAATCGACTTAAAGC.

The *tgl3*Δ */tgl3*Δ diploid strain yWH74 was generated by first transforming yXL004 with YCp50-HO [90] to induce mating type switch and subsequently spontaneous mating of cells in the same colony. Homozygous diploid cells were identified by their inability to mate as either a MATa and MATalpha strain. Diploid cells were then grown in YPD for two days to allow for the loss of YCp50-HO, resulting in 5-FOA resistant, *ura3*^-^ cells.

For growth curve analyses, yeast cells were seeded at an initial concentration of 0.1 OD_600_ in 150 μl of YPD medium in 96-well plates with biological and technical duplicates. The plates were examined by measuring OD_630_ every 30 minutes via a BioTex PowerWave XS plate reader until cells reached saturation. The machine was programmed to shake at the high-speed setting and to control temperature at 30°C.

### Lifespan analyses

Chronological lifespan was measured by the outgrowth method as described in [85]. Briefly, yeast from stab cultures were inoculated to YPD broth and grown at 30°C overnight or until late log phase. Cultures were then diluted into 5 ml SC medium at 0.1 OD_600_. The seeded cultures, in 15-ml glass tubes with a loose metal cap, were incubated in a rotator drum at 30°C. At selected time points, 5 μl of stationary phase cultures were sampled out and mixed with 145 μl of fresh liquid YPD in a 96-well plate. The plates were sealed with parafilm to prevent evaporation, and incubated in the BioTex PowerWave XS plate reader. Cell density (OD_630_) was monitored every 30 minute for 48 hours. Growth curves, doubling times, and survival fractions were calculated according to [85]. To compare the quantitative differences in lifespan of different strains, the time by which each culture reached 10%, 1%, and 0.1% survival fractions was obtained from three independent survival curves. The cultures which survived beyond 30 days before reaching the specified survival fractions were not included in the statistical calculation and represented as >30 days.

For spot assays, stationary phase cultures at selective time points were adjusted to 1 OD_600_ with sterile water in 96-well plates and ten-fold serially diluted. 5 μl of cells from each well were spotted to a YPD plate and grown at 30°C for two days. The culture viability was quantified by counting visible colonies at the most diluted spot. The number of colonies multiplied by the dilution factor of that spot was regarded as the viability. This method was adapted from the Tadpole assay as described in [91].

Replicative lifespan was performed according to previously described [92] by counting number of progeny produced by 60–90 virgin cells from young mother of each strain. Daughter cells were removed every 90 minutes by a micromanipulator. The lifespan analyses were performed by using R software, version 3.0.3 with survival and KMsurv packages. Breslow test was used for statistic analysis.

### Lipid analysis and Nile red staining

Total lipid extraction was conducted essentially as described before [70] with modifications. Briefly, 3 OD_600_ cells from selected time points were harvested by centrifugation at 5,000 rpm for 5 minutes at room temperature, followed by washing once with 1 ml water, and were kept at -80°C if lipid extraction was not done immediately after cell collection. To extract total lipids, cells, if frozen, were removed from the freezer and mixed directly with 300 μl of glass beads (425–600 μm, Sigma-Aldrich) and 1 ml of chloroform: methanol (17:1, v/v) (JT Baker) by vortexing twice for 90 seconds at 4°C. The mixtures were briefly spun and the supernatant was moved to a new glass tube. The remaining cell debris and glass beads were vortexed with an additional 1 ml of chloroform: methanol (2:1, v/v). The supernatant was collected as above and pooled with the previous fraction. 1 ml of 0.2 M phosphoric acid and 1 M KCl, was added to the pooled organic fraction and vortexed vigorously for 30 seconds, and spun at 3,000 rpm, 4°C for 5 minutes to separate the organic and aqueous phases. The chloroform phase at the bottom was collected and dried under nitrogen gas. This lipid extract served as the total lipid fraction. To purify TAG, total lipids were developed by thin-layer chromatography (TLC) on a G60 silica plate (EMD Chemicals). The mobile phase was composed of petroleum ether (35–60°C, Macron): diethyl ether (JT Baker): acetic acid (JT Baker) = 80: 20: 1 (v/v/v). Following development, the TLC plates were briefly stained with iodine vapor to reveal the position of TAG. Spots co-migrating with a TAG control (olive oil, Dante) were isolated and converted to fatty acyl methyl esters (FAME) by reacting with 1 ml of 1 N Methanolic HCl (Sigma-Aldrich) at 80°C for 25 minutes [93]. A universal internal control of 5 μg of pentadecanoic acid (Sigma-Aldrich) was included in all samples for FAME derivatization and gas chromatography (GC). 1 ml of 0.9% NaCl was added to stop the FAME reaction, followed by the addition of 1 ml of hexane, and vortexed for 30 sec to extract FAME. Centrifugation at 3,000 rpm for 5 min at 4°C was conducted before the hexane layer was aspirated to another glass tube, and the volume reduced to 30 μl under nitrogen blowing. 2 μl of the FAME in hexane was injected to a gas chromatography system (Agilent Technology, 7890A) for quantification.

To visualize lipid droplets in yeast cells, approximately 0.5 OD_600_ cells were collected by centrifugation (14,000 rpm for 1 min in a microfuge) and washed once by 1 ml TE buffer (pH 7.4). Cell pellets were suspended in 100 μl of TE buffer and stained with 1 mg/ml Nile red in the presence of 3.7% formaldehyde for concomitant fixation, and let sit in the dark for 20 min. Cells were collected again by microcentrifugation and re-suspended in 100 μl TE buffer and stored in the dark for no more than two days. For microscopy, an Olympus BX51 station with a Exfo X-cite 120 UV light fixture and a DP30-BW CCD camera were used. A GFP filter was used for lipid droplet fluorescence detection. For semi-quantitative comparison of lipid droplets, a fixed exposure (typically 2 seconds) for fluorescence was applied to all samples.

### Sporulation and mating analysis

Sporulation efficiency was determined by microscopically examining the percent of diploid cells forming asci. Overnight YPD cultures of diploid strains were transferred to PSP2 medium (Potassium phthalate (Sigma-Aldrich) 8.3 g/l, yeast extract 1 g/l, 1.7 g/l yeast nitrogen base without amino acids or ammonium sulfate, ammonium sulfate 5 g/l, potassium acetate 10 g/l, pH 5.4 at 0.1 OD_600_ and grown at 30°C under vigorous shaking (200–250 rpm). After 24 hours, cells were harvested by centrifugation (14,000 rpm, 30 sec in a microfuge), washed once with sterile water, and re-suspended in 1 ml SPM medium (potassium acetate 3 g/l, raffinose 0.2 g/l). Cultures were shaken vigorously at 200–250 rpm at 30°C for 48–72 hours. A small amount of cells were removed from the culture, spun, and suspended in 1 mg/ml DAPI (4,6-diamidino-2-phenylindole) in a mounting medium (1 mg/ml p-phenylenediamine, 0.9% glycerol, 2.25 μg/ml DAPI). Percent of cells forming asci with four DAPI foci (i.e., four spores) were counted.

Mating efficiency was quantified by mixing 0.1 OD_600_ of “tested” strains with 0.5 OD_600_ of tester strains of the opposite mating type (227a or 70 ‹), all in early log phase, in 1 ml of YPD. Cell mixtures were let sit at 30°C for 5 hours. The tested and tester stains were also incubated separately as the negative control. Cells were washed once with sterile water and plated on SD medium (2% glucose, 1.7 g/l nitrogen base with amino acids or ammonium sulfate, 5 g/l ammonium sulfate, 20 g/l agar). Mating between the tested and tester strains generated prototroph diploid cells that were able to form colonies on the SD medium plate.

### Wild strains segregation

The strains B454, B756, B779, B359, B370, B357 and B390 were segregants of isolates collected in the wild (see Table 1 for source and reference). In order to generate isogenic haploid strains, the heterozygous wild isolates were sporulated and self diploidized due to homothallism. We randomly selected one of the four homozygous segregants from each tetrad. Next, strains were made heterothallic via removal of the HO gene, using homologous gene replacement with either a Hgh cassette (pAG32) [94] (strain B454 due to a natural kanamycin resistance) or a KanMX6 cassette (pFA6a) [95] (strains B756, B779, B359, B370, B357 and B390). Transformed strains were sporulated and dissected to verify 2:2 segregation of either hygromycin or kanamycin resistance. One MATa and MATalpha haploid transformant was retained from each strain. Proper integration at the *HO* locus was verified via PCR. Strain B653 was obtained from John McCusker.

## Supporting Information

S1 Fig Lab and wild yeast strains used in this work were examined by microscopy. 5-day old stationary phase cultures were harvested for neutral lipid staining with Nile red. DIC (differential interference contrast) and fluorescence microscopy were done using an Olympus BX51 station equipped with a Exfo X-cite 120 UV light fixture and a DP30-BW CCD camera. Fluorescence micrographs were taken with a fixed, 2-second exposure to aid comparison of the fluorescence intensity. Scale bars: 5 μm. All pictures were re-sized identically for presentation.
(TIF)

S2 Fig Deleting either or both TAG lipase genes resulted in similar upregulation of TAG in yeast. Total TAG, expressed as percentage of total cellular lipids, of wildtype, *tgl3*Δ, *tgl4*Δ, and *tgl3*Δ *tgl4*Δ strains were isolated and quantified by gas chromatography. Cells were from 3-day old YPD cultures. *, P<0.05; **, P<0.01
(TIF)

S3 Fig TAG accumulation does not affect mating efficiency, sporulation, or medium acidification. (A) Haploid strains, as indicated, were tested for their mating efficiency with a tester strain. (B) TGL3 +/+ and -/- diploid strains were subjected to sporulation. 3 days after transferring cells to the sporulation medium, cells were examined under a microscope to quantify for the number of tetrads.
(TIF)

S4 Fig TAG-rich and–deficient cells do not exhibit significant differential susceptibility to common stresses. Day 3 early-stationary phase cultures were exposed to the shown stresses before plating to YPD. For the test of salt sensitivity, NaCl (0.7 or 1.4 M) was included in the YPD plate. Heat and H_2_O_2_ sensitivity were conducted by exposing cell suspension (after water wash for H_2_O_2_) to the stress before serially diluted for plating. For UV exposure, serially diluted cells were spotted to YPD before UV exposure. The plates were incubated in dark afterwards.
(TIF)

S5 Fig Deleting *TGL3* does not rescue the early death phenotype of the *dga1*Δ *lro1*Δ lean cells. The indicated strains (on the right) were grown in SC with 2% glucose and sampled at the indicated time for semi-quantitative comparison of colony forming units. 10-fold serially diluted cell suspension was spotted to YPD.
(TIFF)

S6 Fig Combining *tgl3*Δ and *sch9*Δ null alleles caused stochastic growth defects. *SCH9* was deleted from *TGL3*^+^ and *tgl3*Δ backgrounds for chronological lifespan assessment. Two independent *tgl3*Δ *sch9*Δ transformation colonies were isolated and tested. Both were ›+, but one apparently was synthetic sick while the other exhibited normal growth and extended lifespan. Shown are representative results of two independent *SCH9* knockout attempts.
(TIFF)
