# Supplementary figures and images for: An Energy-Independent Pro-longevity Function of Triacylglycerol in Yeast
Source: PLoS Genet. 2016 Feb 23;12(2):e1005878. doi: 10.1371/journal.pgen.1005878 (PMC4764362; doi:10.1371/journal.pgen.1005878)

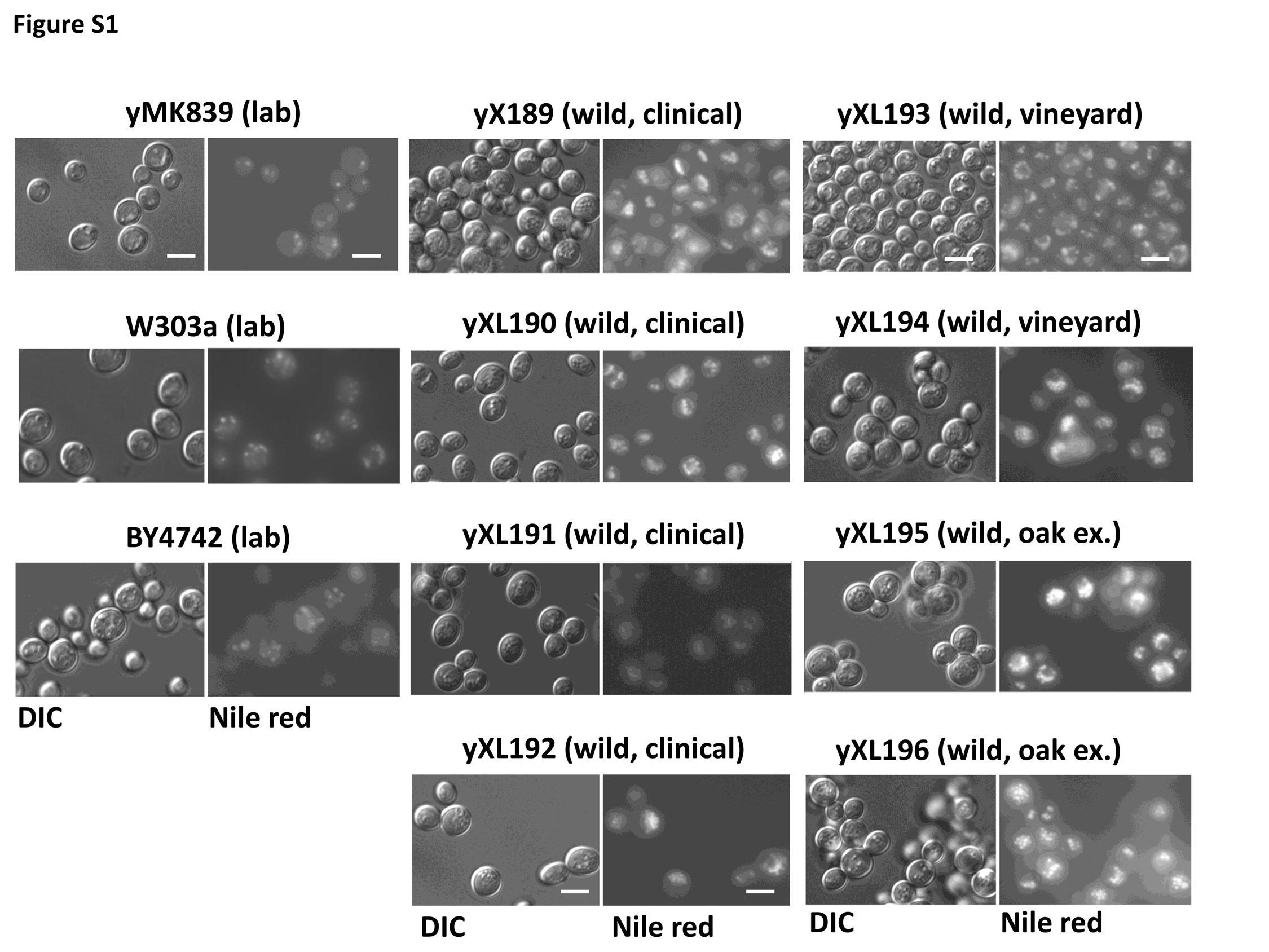

Supplement: S1 Fig — 5-day old stationary phase cultures were harvested for neutral lipid staining with Nile red. DIC (differential interference contrast) and fluorescence microscopy were done using an Olympus BX51 station equipped with a Exfo X-cite 120 UV light fixture and a DP30-BW CCD camera. Fluorescence micrographs were taken with a fixed, 2-second exposure to aid comparison of the fluorescence intensity. Scale bars: 5 μm. All pictures were re-sized identically for presentation. (TIF) [file pgen.1005878.s001.tif]

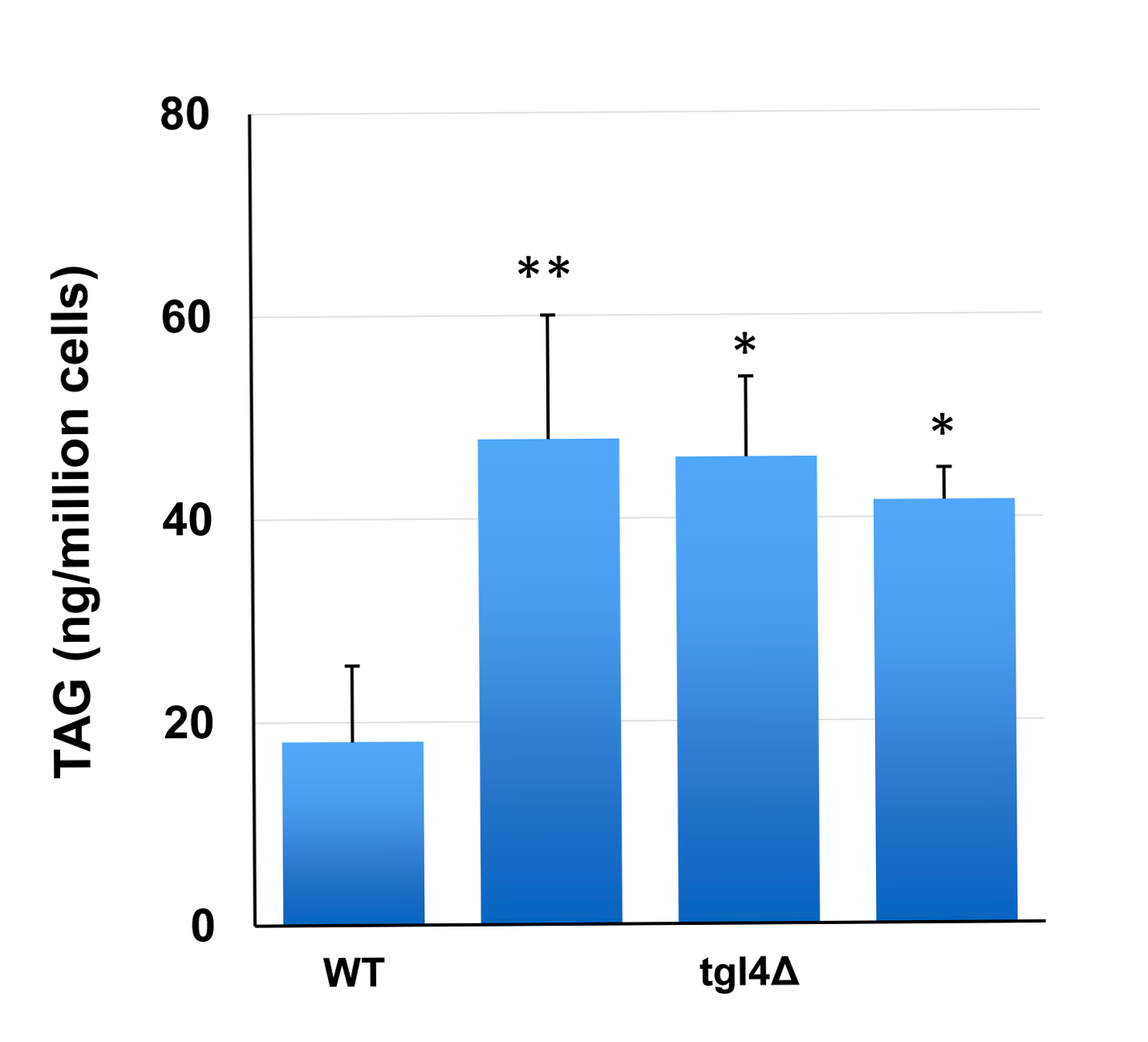

Supplement: S2 Fig — Total TAG, expressed as percentage of total cellular lipids, of wildtype, tgl3Δ, tgl4Δ, and tgl3Δ tgl4Δ strains were isolated and quantified by gas chromatography. Cells were from 3-day old YPD cultures. *, P<0.05; **, P<0.01 (TIF) [file pgen.1005878.s002.tif]

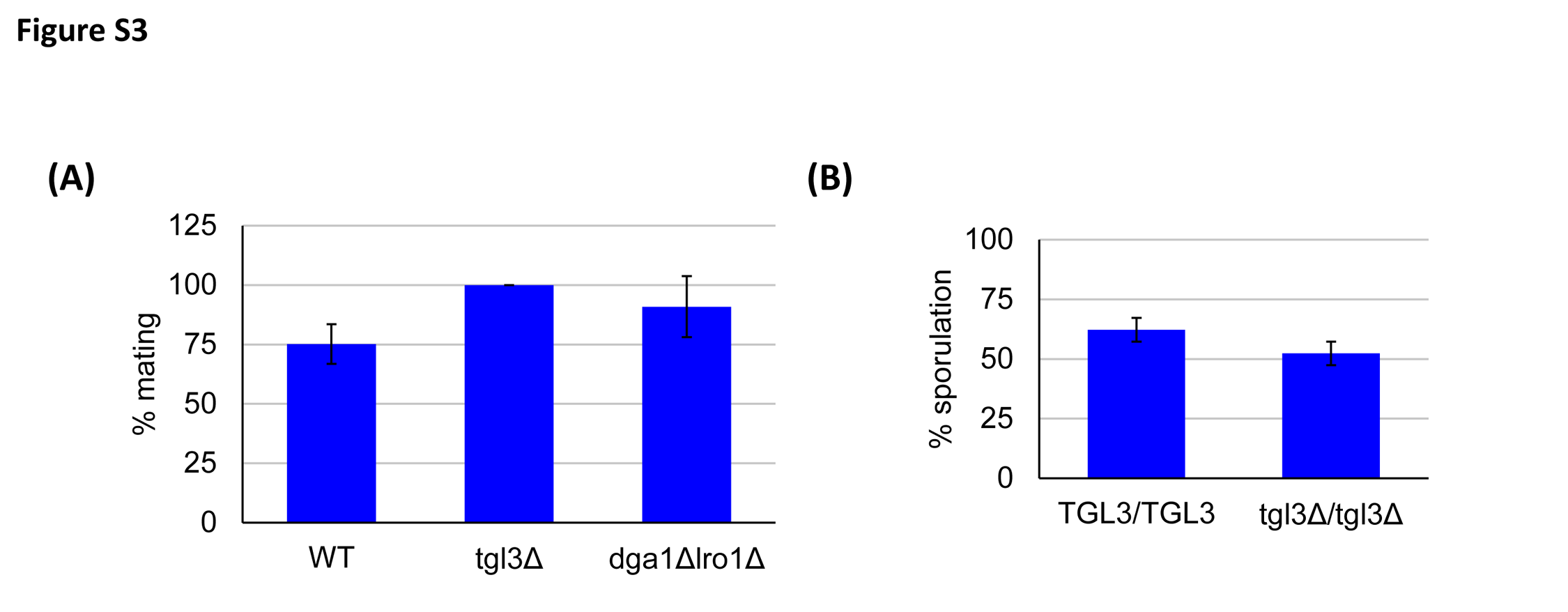

Supplement: S3 Fig — (A) Haploid strains, as indicated, were tested for their mating efficiency with a tester strain. (B) TGL3 +/+ and -/- diploid strains were subjected to sporulation. 3 days after transferring cells to the sporulation medium, cells were examined under a microscope to quantify for the number of tetrads. (TIF) [file pgen.1005878.s003.tif]

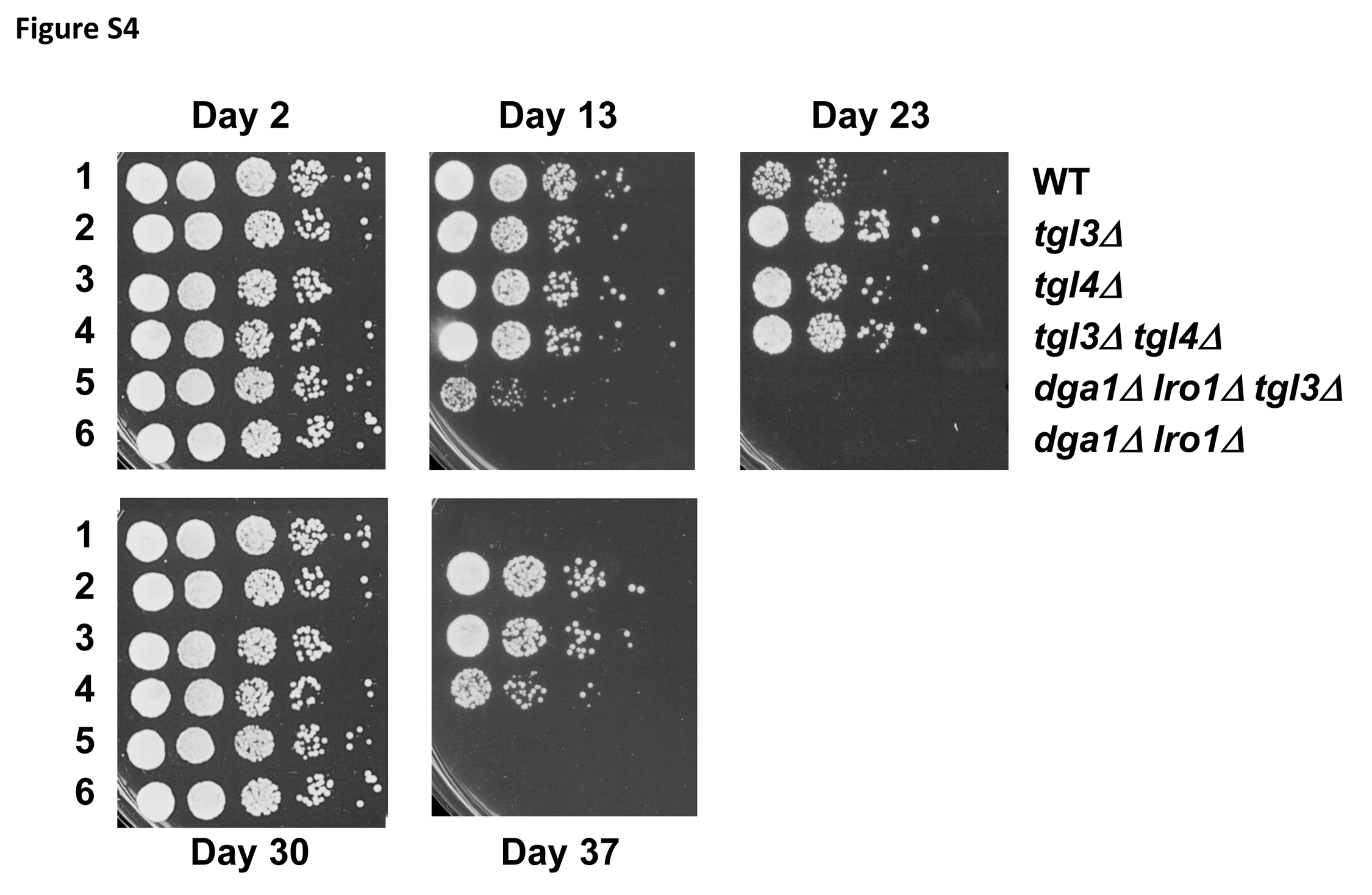

Supplement: S4 Fig — Day 3 early-stationary phase cultures were exposed to the shown stresses before plating to YPD. For the test of salt sensitivity, NaCl (0.7 or 1.4 M) was included in the YPD plate. Heat and H2O2 sensitivity were conducted by exposing cell suspension (after water wash for H2O2) to the stress before serially diluted for plating. For UV exposure, serially diluted cells were spotted to YPD before UV exposure. The plates were incubated in dark afterwards. (TIF) [file pgen.1005878.s004.tif]

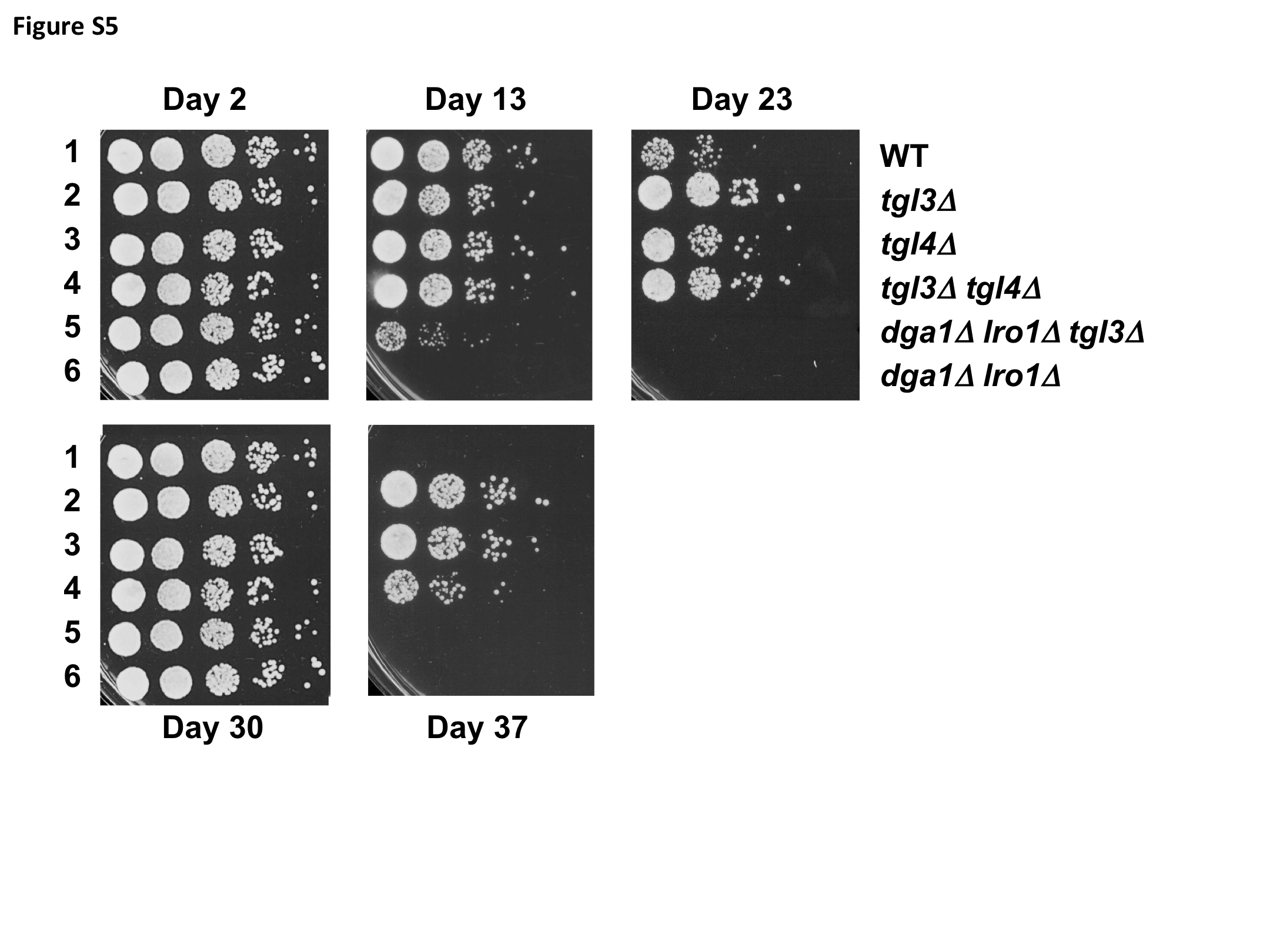

Supplement: S5 Fig — The indicated strains (on the right) were grown in SC with 2% glucose and sampled at the indicated time for semi-quantitative comparison of colony forming units. 10-fold serially diluted cell suspension was spotted to YPD. (TIFF) [file pgen.1005878.s005.tiff]

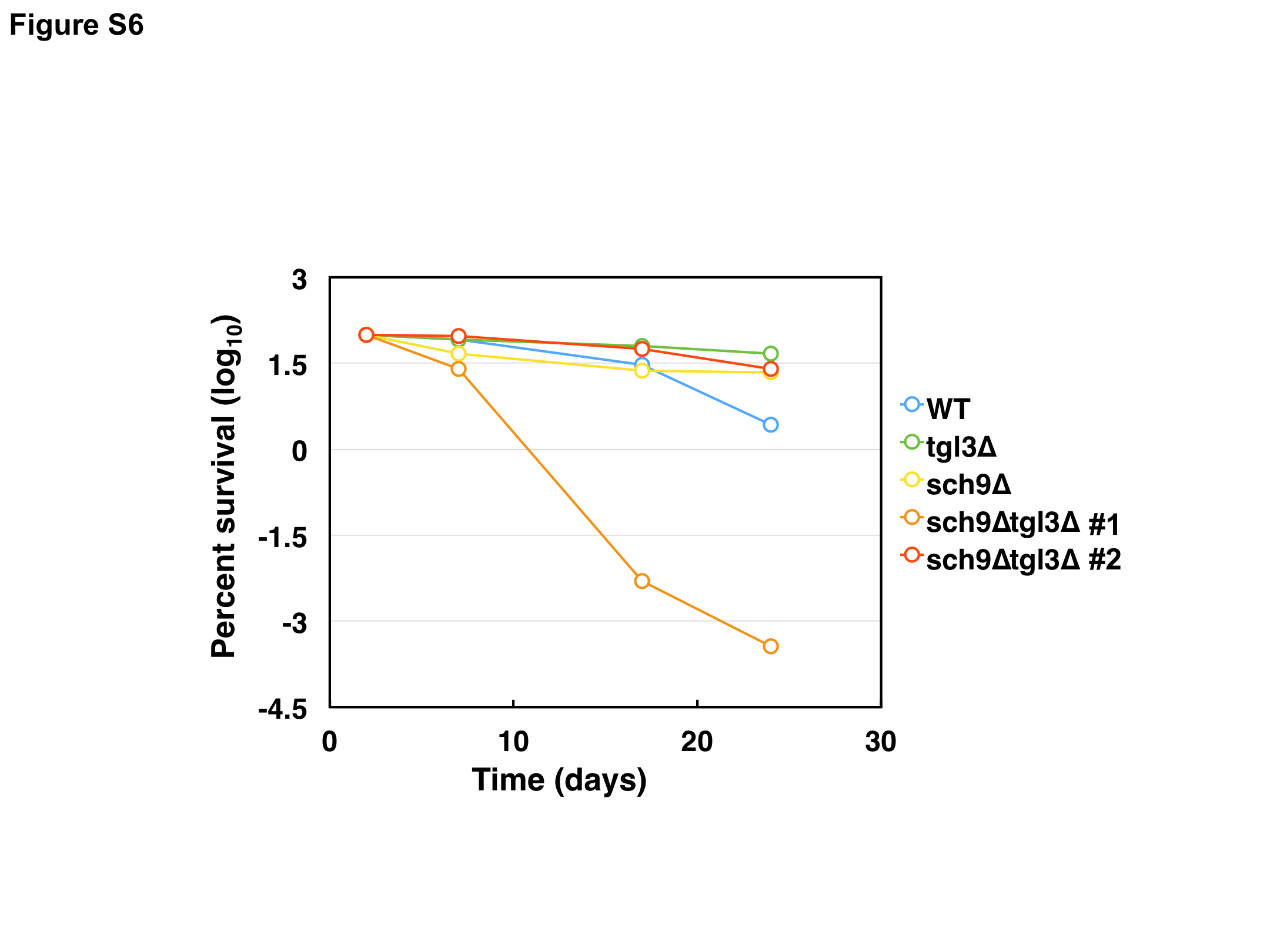

Supplement: S6 Fig — SCH9 was deleted from TGL3+ and tgl3Δ backgrounds for chronological lifespan assessment. Two independent tgl3Δ sch9Δ transformation colonies were isolated and tested. Both were ›+, but one apparently was synthetic sick while the other exhibited normal growth and extended lifespan. Shown are representative results of two independent SCH9 knockout attempts. (TIFF) [file pgen.1005878.s006.tiff]
